# The middle ear-nasopharyngeal microbiome axis associated with obstructive Eustachian tube dysfunction in chronic otitis media

**DOI:** 10.1128/msystems.00007-26

**Published:** 2026-05-19

**Authors:** Xiaoxin Chen, George Gong Chen, Zhongqin Gong, Hengyan Zhu, Ziqi Liang, Jason Ying Kuen Chan, Michael Chi Fai Tong, Zigui Chen, Wai Tsz Chang

**Affiliations:** 1 Department of Otorhinolaryngology, Head and Neck Surgery, The Chinese University of Hong Kong 26451, Hong Kong SAR, China; 2 Department of Pediatric Otolaryngology, Southern Medical University, Shenzhen Hospital, Shenzhen, China; 3 Department of Microbiology, Faculty of Medicine, The Chinese University of Hong Kong 26451, Hong Kong SAR, China; 4 The Chinese University of Hong Kong, Institute of Human Communicative Research 26451, Hong Kong SAR, China; The University of Hong Kong, Hong Kong, Hong Kong

**Keywords:** human microbiome, 16S rRNA gene sequencing, middle ear, nasopharynx, Eustachian tube dysfunction, chronic otitis media

## Abstract

Obstructive Eustachian tube dysfunction (ETD) commonly complicates chronic otitis media (COM), yet microbial factors at the Eustachian tube (ET) openings remain poorly understood. In this prospective cohort study, we characterized the microbiota at both the middle ear (ME) and nasopharyngeal (NP) ET openings in COM patients undergoing ear surgery and examined associations with obstructive ETD over one year of follow-up. Using 16S rRNA gene sequencing and functional inference, we profiled microbial communities from the ME-side ET opening and the torus tubarius on both surgical-side and contralateral nasopharynx. Among 37 patients (18 with ETD, 19 without), ME and NP microbiota differed significantly in composition. Ears with ETD showed increased ME microbial diversity and enrichment of *Neobacillus*, *Agrobacterium*, and *Sphingomonas*. Paired NP-ME analyses indicated an altered NP–ME microbial relationship in ETD, with *Neobacillus* showing a nasopharyngeal source signal. Functional prediction revealed increased porphyrin metabolism and decreased pyruvate metabolism in ME microbiota with ETD, suggesting a shift toward biofilm formation and altered redox states. *Anaerococcus* was increased in the nasopharynx of patients with bilateral COM. These findings identify distinct microbial and metabolic features of COM with obstructive ETD, supporting a role for nasopharyngeal-microbial influence on ME pathology.

**IMPORTANCE:**

Chronic otitis media (COM) is a common and often persistent ear disease, especially when complicated by Eustachian tube dysfunction (ETD). By profiling microbiota at both Eustachian tube openings, this study links upper-airway microbial ecology with middle-ear microbial states in COM and helps clarify where clinically relevant signals may arise along the Eustachian tube pathway. The paired nasopharyngeal–middle ear design revealed that nasopharyngeal microbes may be linked to middle-ear community shifts in COM with obstructive ETD, consistent with a potential upper airway contribution to the middle-ear microbiota, generating testable hypotheses about microbial exchange and persistence. These findings highlight the upper airway microbiome as a potential target for developing new preventive and therapeutic strategies in COM.

## INTRODUCTION

Chronic otitis media (COM), including chronic suppurative otitis media (CSOM) and cholesteatoma, is a common and often persistent condition that can lead to hearing loss and repeated surgery. Obstructive Eustachian tube dysfunction (ETD) is frequently present in COM and is thought to contribute to poor middle-ear ventilation and clearance (1). However, why some patients develop persistent effusion and chronic disease, while others recover, remains incompletely understood, and the biological links between ETD and the middle-ear environment are still unclear.

The Eustachian tube (ET), referred to as the auditory or pharyngotympanic tube, is a fibrocartilaginous structure that links the middle ear to the nasopharynx. Normal ET functions include equalizing air pressure, facilitating fluid drainage, and providing protection against pathogens (1, 2). Obstructive ETD is developed when the Eustachian tube is blocked due to factors such as inflammation, tumors, structural abnormalities, muscular weakness, or damage resulting from radiotherapy (3). The common causes of ET inflammation are bacterial infections and biofilms originating from the nasal cavity and sinuses (4). The nasopharynx is a major microbial reservoir of the upper airway, and it has long been considered relevant to otitis media (5). Classical otopathogens such as *Streptococcus pneumoniae*, *Haemophilus influenzae*, and *Moraxella catarrhalis* are carried in the nasopharynx and have been implicated in middle-ear infection (6, 7). More recent studies further suggest that microbial communities differ across the nasopharynx, middle ear, and external ear canal, and that chronic disease can involve polymicrobial communities and biofilm-associated states (8, 9). Furthermore, evidence has indicated an overlap of the middle-ear microbiome among acute otitis media (AOM), otitis media with effusion (OME), and COM. This suggests the potential existence of the microbial continuum, progressing from AOM caused by infection with a single species to polymicrobial COM as a secondary infection (2, 10–12).

Despite this progress, the relevance of ET microbiome to ETD and COM remains uncertain. A major limitation is that studies sample only one site or one side and cannot evaluate the ET as a potential pathway linking the upper airway and middle-ear microbiota (13). Another limitation is the lack of a consistent, COM-specific measure of obstructive ETD, making it difficult to test whether particular microbial patterns are associated with ETD status and subsequent outcomes.

We previously developed an effective diagnostic model to identify obstructive ETD in patients with COM (14). Based on this diagnostic tool, our current study aimed to profile the microbial community of the ET in patients with COM and to identify the bacterial features of obstructive ETD, as well as to demonstrate the correlation between microbiome and the clinical outcome of COM. Our findings could provide a specific scope for the role of ET microbiome in the pathogenesis of ETD and COM, potentially informing the development of novel therapeutic approaches for ETD, such as personalized probiotic formulations and innovative anti-biofilm agents.

## MATERIALS AND METHODS

### Study design and subjects

This prospective cohort study was conducted at the Department of Otorhinolaryngology, Head and Neck Surgery, within the New Territories East Cluster (NTEC) of the Hong Kong Hospital Authority from February 2023 to December 2024. Patients diagnosed with chronic otitis media (COM), including subtypes of chronic suppurative otitis media (CSOM) and cholesteatoma, who were scheduled for operative treatments, were prospectively recruited. CSOM was diagnosed based on the presence of otorrhea and hearing loss, with a perforation of the tympanic membrane noted during otoscopic assessment. The presence of cholesteatoma was identified through otoscopic examination and further confirmed by high-resolution computed tomography (CT) or magnetic resonance imaging (MRI) (15).

### Inclusion and exclusion criteria

Inclusion criteria were as follows: (i) age between 18 and 70 years old and (ii) requirement for otologic operations with signed informed consent. Exclusion criteria included (i) concomitant external or inner ear diseases, (ii) patulous Eustachian tube dysfunction, (iii) temporomandibular joint disorders, (iv) upper respiratory tract infection within 2 weeks prior to surgery, (v) cleft lip and/or palate; (vi) use of topical or systemic antibiotics within 2 weeks before surgery, and (vii) topical nasal sprays (including intranasal corticosteroids, antihistamines, and decongestants) were withheld for at least 1 week prior to surgery.

### Diagnostic criteria

Patients with COM were classified as an obstructive ETD group if the participants met both of the following conditions (14): (i) a score of 5 or lower on the 5-item Eustachian tube score (ETS-5), which integrates objective tubomanometry findings with subjective feedback related to swallowing and the Valsalva maneuver (16); and (ii) pharyngeal mucosal inflammation around the orifice of the Eustachian tube graded as mild edema or higher on nasal endoscopy (17), using the classification by Poe et al., which categorizes nasopharyngeal conditions into four distinct classes, including normal, mild edema or erythema, moderate inflammation with impaired dilation, and severe inflammation with lumen occlusion (18). It is worth noting that patulous ETD was excluded based on the presence of symptoms such as autophony, fluctuating ear fullness, improvement when lying down, and fluctuating middle-ear pressure linked to breathing, as evidenced by tympanometry or tubomanometry (19). Participants were categorized into two groups: COM with ETD and COM without ETD. All patients were followed up for 1 year postoperatively to monitor COM recurrence, defined as tympanic membrane reperforation, recurrent middle-ear infection, and/or cholesteatoma development at 1-year follow-up.

### Sample size and power considerations

An *a priori* sample size calculation was not performed because effect sizes and variance for the primary multivariate microbiome endpoints in COM patients with or without ETD were not available in the literature, particularly for paired middle-ear and nasopharyngeal Eustachian tube opening sampling. Sample size was determined by patient availability and intraoperative sampling feasibility. The study should be interpreted as exploratory.

### Clinical characteristics collection

Demographic information (age, gender, smoking, drinking, diabetes, and hypertension) and clinical features (affected side, allergic rhinitis [AR], chronic rhinitis or rhinosinusitis, asthma, and laryngopharyngeal reflux symptoms) were recorded during enrollment. Nasal endoscopy, otoscopy, tubomanometry (TMM), tympanometry, and ETS-5 assessments were performed by trained clinicians and audiologists. Additionally, patient-reported symptomatic outcomes were collected using validated questionnaires, including the 7-item Eustachian tube dysfunction questionnaire (ETDQ-7) score for ETD (20) and the 22-item sino-nasal outcome test (SNOT-22) score for nasal disease (21).

### Sample collection, processing, and DNA extraction

Sterile swabs (FLOQSwabs 501CS01, Copan) were used to collect samples from both the middle ear and nasopharyngeal opening of the Eustachian tube during the surgery. To minimize contamination, swabs were carefully maneuvered to avoid contact with extraneous sites such as the external auditory canal or nasal cavity. Each swab was rotated and rubbed at the Eustachian tube for at least three times to maximize microbial biomass yield. Nasopharyngeal samples were obtained from the surgical side (NS) and the paired non-surgical (control) side (NC) from the same patient. Middle-ear swab samples were collected from the Eustachian tube opening of the middle ear (ME). Swab tips were immediately immersed in 500 µL of transport medium (Remel MicroTest M4RT, Thermo) within a 2 mL tube and transported to the laboratory within 1 h. Upon arriving, tubes were vortexed at low speed and centrifuged at 1,600 × *g* for 10 minutes at 4°C. The supernatant was discarded, and the pellet samples (including the swab) were stored at −80°C until further processing. For participants with bilateral COM, only the operated ear was included as the index ear for analysis to avoid non-independence across ears within the same individual.

### 16S rRNA gene V3–V4 amplification and sequencing

Total genomic DNA was extracted from the pellets using the Qiagen DNeasy Blood and Tissue Kit (Qiagen, Valencia, CA, USA) following the manufacturer’s protocol. A minor modified primer set targeting the bacterial 16S rRNA gene hypervariable V3–V4 region (341F, 5′-CCT ACG GGN GGC WGC AG-3′; 806R, 5′-GGA CTA CNV GGG TWT CTA AT-3′) was used to PCR-amplify a broad spectrum of human microbiota (22, 23). In brief, a pair of dual unique 12 bp barcodes was indexed to each amplicon set through the forward and reverse primers modified from the Earth Microbiome Project protocol (https://earthmicrobiome.org); successful amplicons were equally pooled and sequenced on an Illumina MiSeq using paired-end 300 bp reads. For quality control, each sequencing batch included a mock community, DNA negative controls, and technical replicate samples (24).

### Data processing and bioinformatics analysis

Demultiplexed short 16S reads passing the quality filter were imported into the QIIME2 package (v2025.7) to generate an amplicon sequence variant (ASV) table as previously described (22, 24). Representative sequences were subsequently classified at the species level utilizing pplacer, which integrates these sequences into a phylogenetic tree constructed from full-length bacterial 16S rRNA gene sequences to optimize phylogenetic likelihood (25). ASVs exhibiting a total count greater than 10, following the exclusion of reads attributed to archaea, mitochondria, or chloroplasts, were retained. Operational taxonomic unit (OTU) count tables were generated to display the abundance of bacterial reads per sample across various taxonomic ranks. A phylogenetic tree was constructed by integrating the representative ASV sequences into the SILVA v138.2 reference database utilizing the SATe-enabled phylogenetic placement (SEPP) approach (26). Alpha diversity of the observed bacterial ASV reads based on richness and Shannon indices was calculated using the Vegan R package. Beta diversity was assessed using unweighted GUniFrac, weighted GUniFrac, and Bray–Curtis distances in the R v3.4.0 package. Differences in community composition were assessed using permutational multivariate analysis of variance (PERMANOVA) in the Vegan package in R. Principal coordinate analysis (PCoA) was performed to visualize associations between community composition. Discriminative bacterial taxa between ETD and non-ETD groups based on OTU count tables were estimated using linear discriminant analysis (LDA) effect size (LEfSe) analysis (27), with a cutoff of LDA > 2 (*q* < 0.05), which was further validated by different compositional-aware tools with bias correction using ANCOM-BC2 (28). Comparisons of the relative abundances of bacterial genera between defined groups were performed using the non-parametric Mann–Whitney Wilcoxon rank sum test (MWU). Spearman’s rank-order correlation test was used to explore the associations between discriminative bacterial taxa. The clinical characteristics were analyzed using SPSS 28.0 (Chicago, IL). A *t*-test was used to compare means, and a chi-square test was utilized to compare categorical variables. A two-sided *P*-value < 0.05 and/or a false discovery rate (FDR)-adjusted *P*-value (*q* value) < 0.05 was used as the threshold for significance.

### Microbial source tracking analysis

To investigate the potential origins of microbial communities associated with ETD and COM, we employed the fast expectation-maximization for microbial source tracking (FEAST) algorithm. FEAST is a probabilistic modeling tool designed to estimate the proportional contributions of predefined source environments to a given microbial community (sink), based on taxonomic profiles (29). In this study, microbial source tracking was performed using ASV-level relative abundance data derived from 16S rRNA gene sequencing. The FEAST algorithm was then applied to quantify the contribution of microbial communities from potential source sites (e.g., nasopharynx) to the middle-ear microbiota. We referred to the FEAST-estimated nasopharyngeal source contribution proportion to middle-ear samples as the nasal-to-ear translocation metric (operational definition), noting that it represents model-based source attribution rather than direct evidence of translocation. The method assumes that the sink microbial community is a mixture of microbes from one or more source environments and computes maximum-likelihood estimates of these contributions using an expectation-maximization (EM) approach. The analysis was carried out using the publicly available FEAST implementation (https://github.com/cozygene/FEAST), with default parameters unless otherwise specified. Statistical significance of source contributions was evaluated using permutation-based significance testing embedded in the FEAST pipeline.

### Functional prediction of 16S rRNA gene-based microbial communities

The functional profiles of microbial communities were inferred using PICRUSt2 (https://github.com/picrust/picrust2/wiki) based on 16S rRNA gene sequence data. These profiles were characterized in terms of Kyoto Encyclopedia of Genes and Genomes (KEGG) Orthology (KO) annotations (30). Differential abundances of KO pathways between conditions were analyzed using the EdgeR package in R using a cutoff of *P*-value < 0.05 and log2fold change (FC) > 0.5 or < −0.5 (31). Functional enrichment analyses were summarized using the clusterProfiler package in R. The workflow from participant enrollment and sampling to 16S rRNA sequencing was shown in Fig. S1. The completed STORMS checklist can be accessed via Figshare (DOI: 10.6084/m9.figshare.31712650).

## RESULTS

### Study subjects and sample sequencing

A total of 37 patients with COM were enrolled in the microbiome analysis, including 18 patients with ETD and 19 without ETD. Each patient contributed at least one sample or a complete set from the Eustachian tube opening of the middle ear (ME swabs), the nasopharynx on the surgical side (NS swabs), and the contralateral nasopharynx as an internal control (NC swabs) during operations. In total, 78 samples were analyzed, including 14 ME swabs (7 ears with ETD and 7 ears without ETD), 33 NS swabs (18 with ETD and 15 without ETD based on the relative ears on the same side), and 31 NC swabs (5 with ETD and 26 without ETD based on the relative ears on the same side) (Fig. S2). Relevant data on the demographics and characteristics of the COM patients were presented in Table S1, showing balanced baseline characteristics across ETD strata. However, chronic rhinosinusitis was substantially more prevalent in ETD patients (72.2% vs 21.1%; *P* = 0.002), and endoscopic grading also indicated more severe inflammation at the Eustachian tube nasopharyngeal orifice (grades II–IV: 83.4% vs 15.8%; *P* = 0.001).

### Microbiota communities between ME and NP samples and between ETD and non-ETD

**Fig 1:**
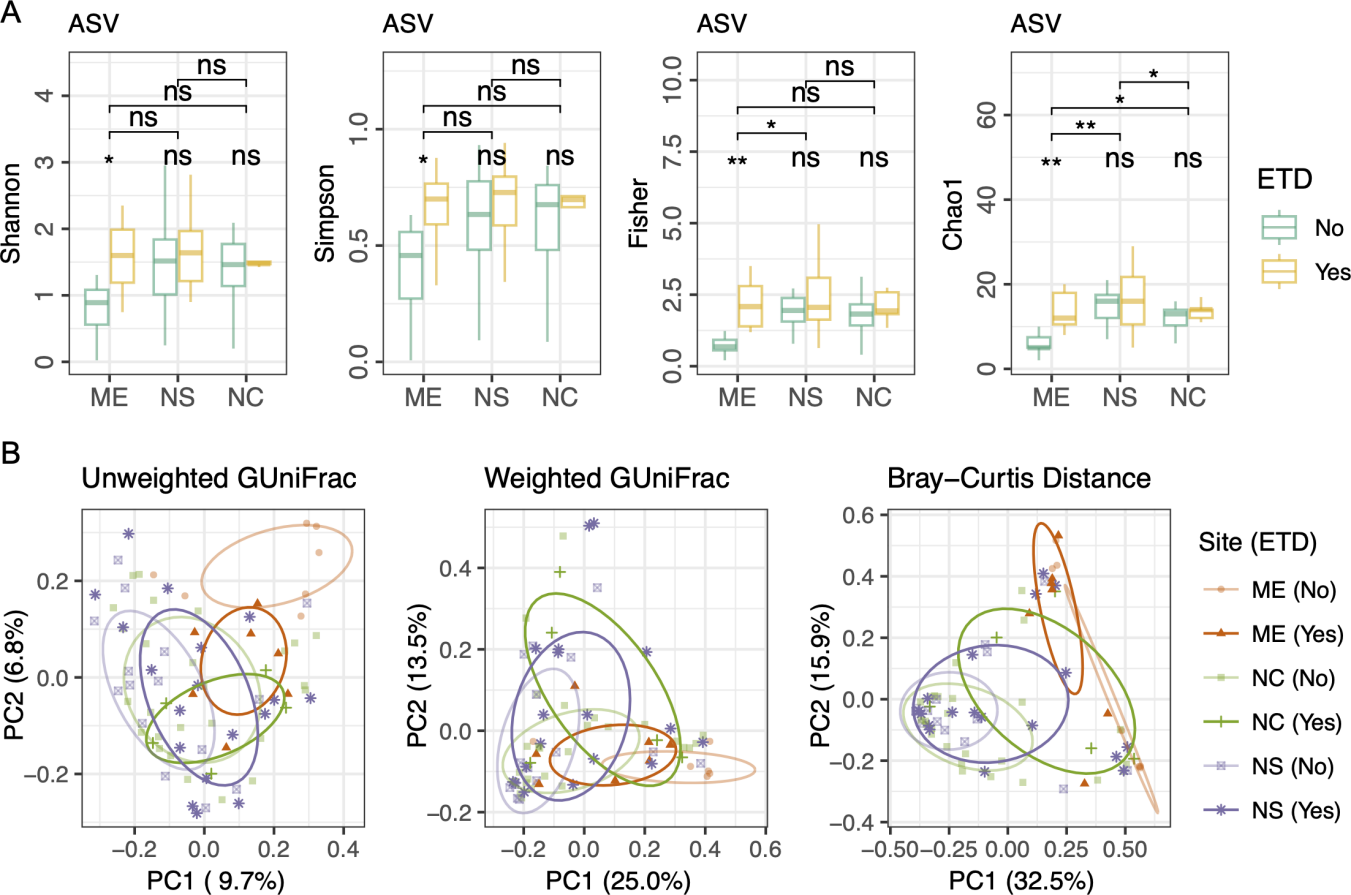
Microbiota diversity of different sites between ETD and non-ETD. (**A**) Comparison of alpha diversity of ME, NS, and NC microbiota between ETD and non-ETD summarized at the genus level. The boxplot’s center line indicates the median value, the box bounds represent the first and third quartiles, and the whiskers extend to the smallest and largest values in the data, respectively. (**B**) Principal coordinates plot using unweighted GUniFrac, weighted GUniFrac, and Bray–Curtis dissimilarity. *P < 0.05, **P < 0.01; ns, not statistically significant.

All samples were characterized for microbial composition using 16S rRNA V3–V4 hypervariable amplification next-generation sequencing. *Actinobacteriota* (50.6% ± 3.7%), *Firmicutes* (34.7% ± 3.5%), *Proteobacteria* (12.2% ± 2.2%), *Bacteroidota* (1.5% ± 0.7%), and *Fusobacteria* (0.5% ± 0.3%) were the five most abundant phyla identified in the overall samples (Fig. S3A; Table S2A). *Actinobacteriota* was the most abundant phylum in both NC (52.4% ± 5.4%) and NS (54.5% ± 5.6%) samples, while *Firmicutes* (55.5% ± 10.6%) appeared to be the most abundant phylum followed by *Actinobacteriota* (37.2% ± 9.7%) in ME samples. When bacterial taxa were summarized at the genus level (Fig. S3B; Table S2B), we found significantly increased alpha diversity of ME microbial communities in ETD samples compared to non-ETD samples (Fig. 1A). Different microbial communities (beta diversity) were also observed between middle-ear and nasopharyngeal samples, using the unweighted GUniFrac, weighted GUniFrac, or Bray–Curtis distances (Fig. 1B; Table S3). However, no significant difference in alpha or beta diversity was observed between the NS and NC microbiota.

### Microbiota dysbiosis is associated with clinical characteristics of COM

**Fig 2:**
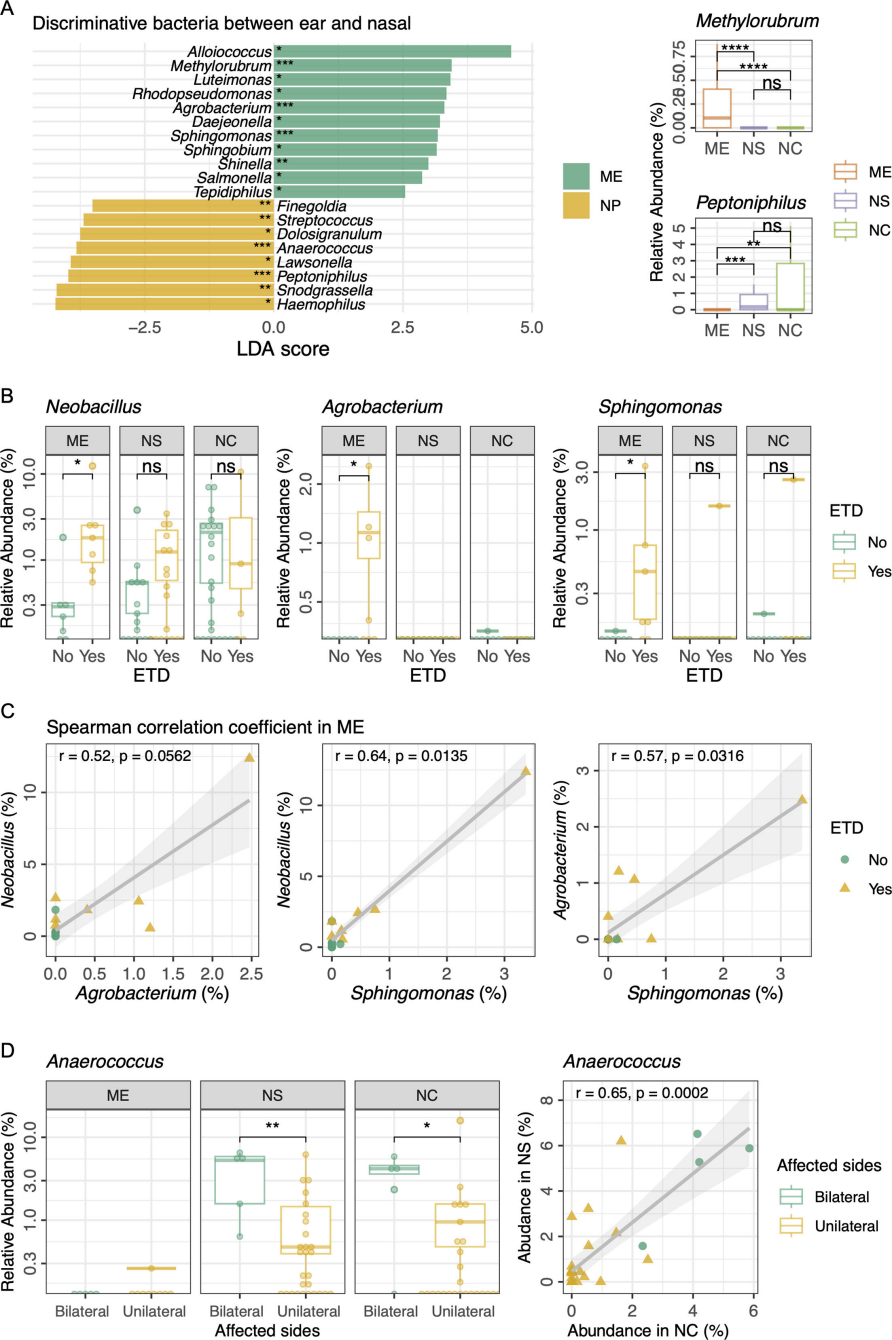
Microbiota dysbiosis associated with clinical characteristics of COM patients. (**A**) Discriminative bacterial genus between middle-ear and nasopharyngeal samples as detected by linear discriminant analysis (LDA) effect size (LEfSe) analysis (score > 3, *q* < 0.05), and the bar length represents log10 LDA score. Differences in the relative abundance of *Methylorubrum* and *Peptoniphilus* between ME, NS, and NC samples were further shown on the right panels. (**B**) Higher abundance of *Neobacillus*, *Agrobacterium,* and *Sphingomonas* in ETD compared with non-ETD in the ME microbiota. (**C**) Positive correlations between *Neobacillus* and *Sphingomonas*, *Sphingomonas* and *Agrobacterium*, associated with ETD in ME samples. (**D**) Higher abundance of *Anaerococcus* in bilateral COM compared with unilateral COM in both the NC and NS microbiota. The abundance of *Anaerococcus* in the NC and NS microbiota associated with the affected sides of COM was positively correlated. *P < 0.05; **P < 0.01; ***P < 0.001; ****P < 0.0001; ns, not statistically significant.

Using a linear discriminant analysis effect size (LEfSe) test (LDA > 2, *q* < 0.05), which was further verified by ANCOM-BC2, we were able to distinguish bacteria between the middle ear and nasopharynx. In total, there were 11 significantly enriched and 8 depleted bacterial genera in the middle-ear samples compared to the nasopharyngeal samples (Fig. 2A; Table S4). For example, the relative abundance of *Methylorubrum*, which was found to be enriched, was significantly greater in ME samples compared to both NS (0.46 ± 0.24 vs 0.00 ± 0.00, *P* < 0.0001) and NC (0.46 ± 0.24 vs 0.02 ± 0.02, *P* < 0.0001) samples. While the relative abundance of *Peptoniphilus*, identified as depleted, was significantly lower in ME samples relative to NS (0.00 ± 0.00 vs 1.95 ± 0.82, *P* < 0.001) and NC (0.00 ± 0.00 vs 1.79 ± 0.52, *P* < 0.01) samples (Fig. 2A), indicating a distinct microbiome environment between the middle ear and the nasopharynx.

In particular, we divided the surveyed samples into ETD and non-ETD groups and found significantly higher abundance of *Neobacillus* (*P* = 0.011), *Agrobacterium* (*P* = 0.031), and *Sphingomonas* (*P* = 0.024) in ETD compared with non-ETD in the ME microbiota, while no significance in the NS and NC microbiota (Fig. 2B). Moreover, significant positive correlations were observed between *Neobacillus* and *Sphingomonas*, as well as between *Sphingomonas* and *Agrobacterium*, in ME samples (Fig. 2C). These associations were linked to ETD, suggesting a potential role of dysfunctional-enriched bacteria in the pathogenesis of ETD. We also observed a greater abundance of *Anaerococcus* in bilateral COM compared with unilateral COM within both the NC and NS microbiota (Fig. 2D). Furthermore, the abundance of *Anaerococcus* in the NC and NS microbiota related to the affected sides of COM demonstrated a positive correlation, indicating that *Anaerococcus* may contribute to the pathogenesis of bilateral disease development.

### FEAST-estimated nasopharynx-to-middle ear microbial source contribution is associated with ETD and elevated *Neobacillus* abundance

**Fig 3:**
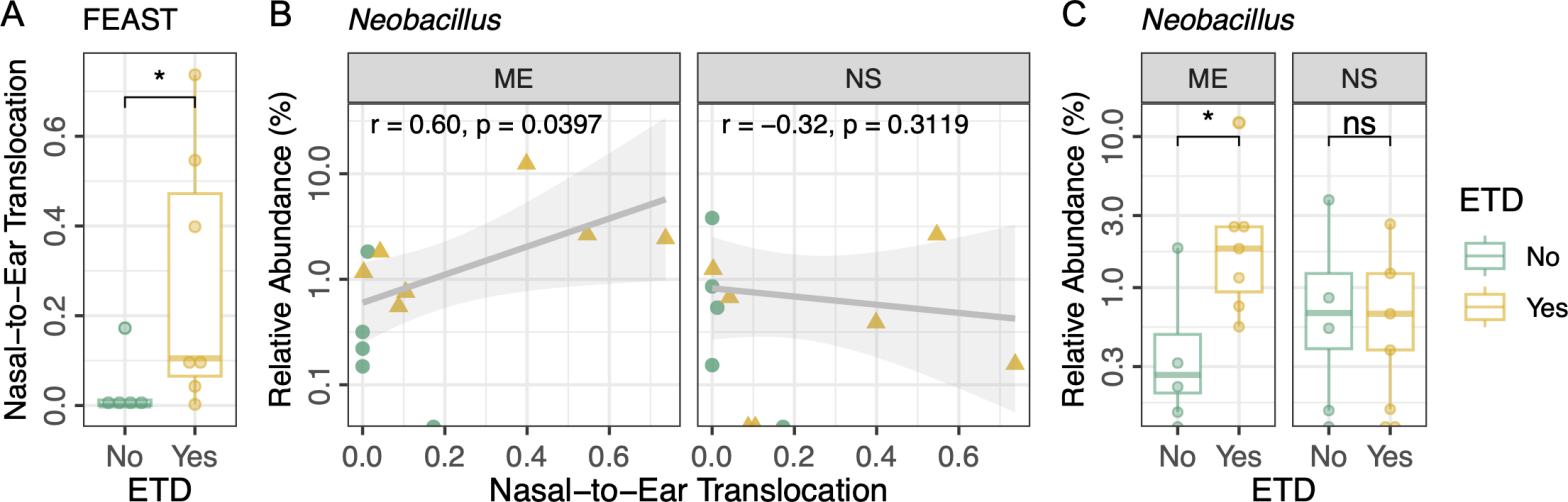
The association between nasopharyngeal source contribution proportion to middle-ear samples, ETD, and the relative abundance of *Neobacillus* using fast expectation-maximization microbial source tracking (FEAST) microbial source tracking tool. (**A**) Higher proportion of nasal-to-ear translocation was positively associated with ETD. (**B**) The abundance of *Neobacillus* was positively correlated to nasal-to-ear translocation in ME samples. (**C**) Higher abundance of *Neobacillus* was positively associated with ETD in ME samples. *P < 0.05; ns, not statistically significant. Nasal-to-ear translocation denotes the FEAST-estimated nasopharyngeal source contribution proportion to middle-ear samples and does not directly demonstrate physical translocation or directionality.

By employing the fast expectation-maximization microbial source tracking (FEAST), we estimated the relative contributions of candidate source communities to the middle-ear microbiome, providing an inferential framework for potential nasopharyngeal-to-middle ear associations. We found a higher proportion of nasal-to-ear translocation was positively associated with the presence of ETD (*P* = 0.0426), indicating that ETD may be associated with an increased nasopharyngeal source signal in the middle ear (Fig. 3A). This finding is further supported by the close clustering observed between ETD groups from the ME and NS samples, as determined by unweighted GUniFrac, weighted GUniFrac, and Bray–Curtis distance metrics (*P* < 0.05; Fig. S4). Meanwhile, the abundance of *Neobacillus* exhibited a positive correlation with nasal-to-ear translocation in ME samples (*r* = 0.60, *P* = 0.0397), whereas no significant correlation was detected in NS samples, suggesting that *Neobacillus* is linked to this source tracking signal in the middle ear (Fig. 3B). Moreover, a higher abundance of *Neobacillus* was significantly associated with ETD (*P* = 0.0315) (Fig. 3C). Collectively, these results suggest that ETD is associated with a stronger nasopharynx-to-middle ear microbial connection and elevated *Neobacillus* in the middle ear. However, direct evidence for nasopharynx-to-middle ear translocation, directionality, or causality will require longitudinal and strain-resolved validation.

### Functional alterations in the middle-ear microbiome associated with ETD

**Fig 4:**
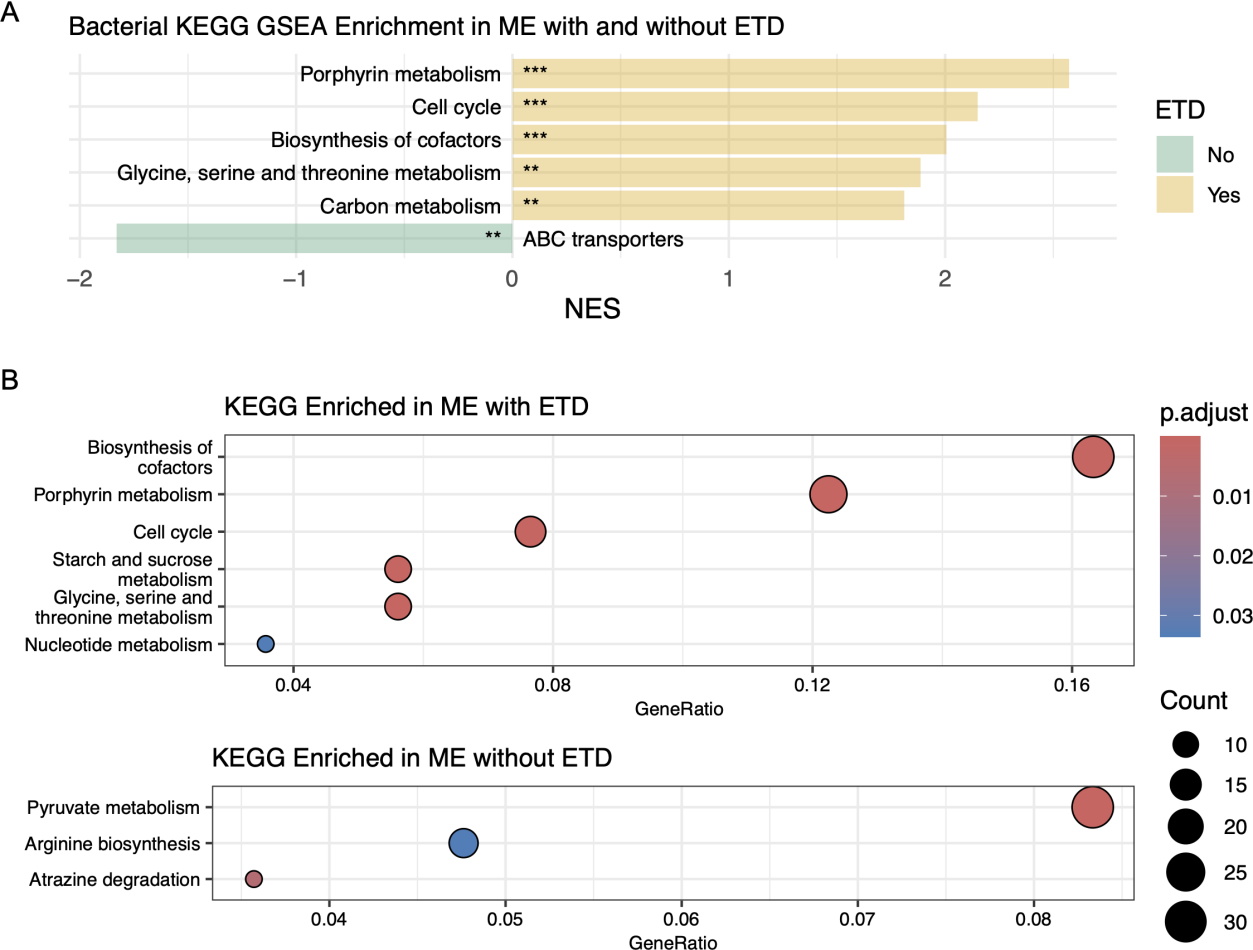
Kyoto Encyclopedia of Genes and Genomes (KEGG) pathways enrichment analysis between ETD and non-ETD in ME samples. (**A**) KEGG pathway enrichment analysis comparing ME microbiota between subjects with and without ETD using gene set enrichment analysis (GSEA). Normalized enrichment scores (NES) reveal upregulation of pathways such as porphyrin metabolism, cell cycle, biosynthesis of cofactors, glycine, serine, and threonine metabolism, and carbon metabolism in the ETD group, and downregulation of pathways including ABC transporters in the non-ETD group. (**B**) Bar plots showing KEGG pathways significantly enriched in ME samples with ETD (up) and without ETD (down), based on gene ratio and adjusted *P*-values. **P < 0.01; ***P < 0.001.

To investigate the functional potential of the middle-ear microbiome and its association with ETD, we performed Kyoto Encyclopedia of Genes and Genomes (KEGG)-based functional enrichment analyses using both gene set enrichment analysis (GSEA) and pathway-level comparisons. Multivariate analysis using sparse partial least squares discriminant analysis (sPLS-DA) was performed to assess beta diversity across anatomical sites and ETD conditions. The resulting ordination plots demonstrated clear separation between sites and partial clustering by ETD status, indicating that both anatomical origin and ETD influence the functional configuration of microbial communities (Fig. S5A). To further characterize overall functional patterns, we conducted a broader GSEA on ranked gene features in ME samples, revealing several microbial metabolic pathways significantly enriched in the ME microbiota of individuals with ETD compared to those without. Specifically, pathways such as porphyrin metabolism, cell cycle, biosynthesis of cofactors, glycine, serine, and threonine metabolism, carbon metabolism, and ABC transporters showed significantly higher normalized enrichment scores (NES) in the ETD group, suggesting increased microbial activity and potential adaptation to a dysregulated middle-ear environment (Fig. 4A; Fig. S5B). In contrast, the non-ETD group showed higher enrichment in pathways such as pyruvate metabolism, arginine biosynthesis, and atrazine degradation, indicating a more homeostatic or less dysbiotic microbial functional profile (Fig. 4B). These results suggest that the presence of ETD is associated with a functional shift in the resident microbiota, potentially reflecting altered host-microbe interactions or environmental pressures within the middle-ear space.

## DISCUSSION

We profiled the microbiota at the ET openings in COM patients and identified bacterial signatures associated with obstructive ETD for the first time. We observed significantly different microbial communities between middle ear (ME) and nasopharyngeal (NP) samples as well as differences between with and without ETD. Source tracking analyses further suggested a stronger NP to ME source signal in ETD patients, and *Neobacillus* was positively associated with this signal in ME samples and enriched in ETD patients. Furthermore, functional analysis suggested enriched porphyrin metabolism and depressed pyruvate metabolism pathways in the ME microbiome of ETD patients. Together, these findings motivate mechanistic studies to test causality and evaluate whether modulating nasopharyngeal reservoirs or ME biofilm propensity can reduce the risk or severity of COM, particularly in patients with ETD..

In the study, both alpha and beta diversity findings indicated a differential microbiome between middle-ear and nasopharyngeal samples, consistent with previous studies (6, 10). *Actinobacteriota*, *Firmicutes*, *Proteobacteria*, and *Bacteroidota* were the most common phyla, while *Corynebacterium* and *Staphylococcus* were the most prevalent genera found in both the middle-ear and nasopharyngeal samples. However, at the genus level, *Haemophilus*, *Peptoniphilus*, *Dolosigranulum*, and *Streptococcus* were absent from the middle-ear samples. This finding contrasts with other studies that demonstrate *Haemophilus* and *Streptococcus* as classic ear pathogens, typically present in high abundance in the middle ear of individuals with otitis media with effusion or acute otitis media (6, 7, 32). A change in the microbiome may occur as middle-ear disease progresses to a chronic and more severe state, such as chronic suppurative otitis media and cholesteatoma.

We also observed taxa that appeared site- or phenotype-enriched. *Alloiococcus* was identified significantly higher in the middle-ear samples but was almost absent in the nasopharyngeal samples, which is consistent with previous studies (5, 7). It has been frequently detected as a dominant or co-dominant taxon in culture-negative COM and has been shown to persist in biofilm communities on the middle-ear mucosa (33, 34). These observations suggest that *Alloiococcus* may serve as a marker of middle-ear dysbiosis or biofilm-associated states in COM, motivating longitudinal studies to evaluate its temporal dynamics and to test anti-biofilm or microbiome-modulating strategies in mechanistic and clinical settings (35). In ETD subjects, *Neobacillus*, *Agrobacterium*, and *Sphingomonas* showed higher relative abundance, suggesting that ETD is associated with a distinct ME microbial profile. *Neobacillus*, a reclassified genus previously grouped within *Bacillus*, has been increasingly recognized in mucosal microbiomes, though its functional role remains largely unexplored (36). *Agrobacterium* and *Sphingomonas* are environmental or opportunistic genera that have been detected in various human mucosal niches, including the respiratory tract and nasopharynx (37). While causality cannot be inferred from this cross-sectional 16S data set, these taxa represent plausible candidates linked to ETD-associated COM and warrant validation in larger cohorts and mechanistic studies.

To explore the role of these opportunistic bacteria in the relationship between ETD and COM, paired ME and NP samples were analyzed using source tracking. We identified an aberrant migration pattern from the nasopharynx to the middle ear, with *Neobacillus* exhibiting a strong signature of potential nasopharyngeal origin and significant enrichment in the middle-ear compartment of ETD subjects. Notably, FEAST estimates are conditional on the sources included in the model and are limited by the taxonomic resolution of 16S V3–V4 data. This source signature is inferential and should be interpreted as hypothesis-generating rather than direct evidence of physical translocation. Mechanistically, the Eustachian tube functions as both a mechanical and immunological barrier, restricting passive movement of microorganisms from the nasopharynx to the sterile middle-ear cavity (38). However, in ETD, impaired mucociliary clearance, altered pressure regulation, and compromised epithelial integrity may facilitate ascending microbial translocation (39). In this context, the enrichment of *Neobacillus* in the middle ear of ETD subjects may reflect putative nasopharyngeal seeding or selective persistence in an altered niche, consistent with reports that commensal-origin taxa can display pathobiont-like behavior after ecological displacement (40, 41), while mechanistic links remain to be validated.

Notably, functional inference of the middle-ear microbiome in patients with ETD revealed a consistent enrichment of porphyrin metabolism and suppression of pyruvate metabolism pathways. These metabolic alterations are indicative of a microbial community undergoing redox stress adaptation and transitioning toward a biofilm-dominant state (42). Porphyrin biosynthesis is closely linked to oxidative metabolism, heme production, and microbial stress responses and has been implicated in the persistence of biofilm-forming bacteria, particularly in chronic mucosal infections (43, 44). The concurrent decrease in pyruvate metabolism reflects either suppressed aerobic energy production or a shift toward fermentation-based survival strategies in a hypoxic or inflamed microenvironment, both of which are hallmarks of the middle-ear cavity during ETD (45). These findings support the hypothesis that the middle-ear microbiota in ETD is functionally reprogrammed toward a low-energy, stress-tolerant, and potentially antibiotic-resistant state (46, 47). Given the concurrent enrichment of *Neobacillus* and its nasopharyngeal source signature, *Neobacillus* may be linked to this predicted functional milieu, although causality and functional activity require validation.

In the nasopharyngeal microbiota, we observed a significant increase in the genus *Anaerococcus* in patients with bilateral COM. *Anaerococcus* is a Gram-positive anaerobic coccus commonly found in the oropharynx, urogenital tract, and skin. While often regarded as a commensal, it has been implicated in polymicrobial infections and abscess formation (48). Its enrichment in bilateral COM may reflect a distinct nasopharyngeal community state associated with more extensive disease (49).

To aid interpretation and reproducibility, we summarized key design considerations, potential confounding, and priorities for future validation. First, this exploratory study (*n* = 37) was constrained by the feasibility of paired intraoperative sampling. A prospective power calculation was not performed because effect-size estimates for key COM and ETD microbiome endpoints are not well established. The study may therefore be underpowered for modest effects, although the paired design reduces inter-individual variability and provides preliminary effect-size estimates for future studies. Second, to avoid non-independence, only the operated ear was analyzed in bilateral COM cases. NS and NC were defined relative to that ear; not every participant contributed a complete triad, explaining the unequal sample counts across sites (14 ME, 33 NS, 31 NC). Because the study was not powered to detect subtle laterality effects, we avoid laterality-specific conclusions. Besides, our sampling did not include the external auditory canal (EAC) or anterior nasal cavity as additional comparator sites. The absence of these site controls limits our ability to more comprehensively account for potential contamination from the ear canal during middle-ear sampling and to evaluate alternative upper-airway reservoirs beyond the nasopharynx. Future studies with multi-site sampling, ideally with paired negative controls, would strengthen source attribution and improve robustness. Third, potential confounding should be considered. Chronic rhinosinusitis and nasal medications may influence nasopharyngeal communities. Sprays were withheld for 1 week preoperatively, but longer-term effects cannot be excluded, motivating prospective cohorts with systematic exposure capture and covariate-adjusted analyses. Fourth, the lack of otologically healthy controls and the cross-sectional surgical sampling limit attribution and causal inference. Future work should include appropriate control groups such as individuals undergoing cochlear implantation or other otologic procedures without COM and longitudinal sampling. Lastly, FEAST and PICRUSt2 results are inference-based and limited by 16S V3–V4 resolution and therefore require replication and strain- or activity-resolved validation, alongside mechanistic testing in middle-ear epithelial co-culture or biofilm models under reduced-aeration conditions with host-response readouts.

### Conclusions

In conclusion, the middle-ear microbiome in COM with obstructive ETD is characterized by greater diversity, enrichment of opportunistic bacteria, and FEAST-based signals consistent with increased nasopharyngeal contribution to middle-ear communities. Predicted functional profiles further indicate ETD-associated shifts in metabolic potential, nominating specific taxa and pathways for targeted longitudinal and mechanistic validation.

## Data Availability

All sequences and codes are available upon request. The 16S rRNA gene amplicon next-generation sequences analyzed in this study are available in the NCBI SRA database under PRJNA1314944.

## SUPPLEMENTAL MATERIAL

Supplemental figures msystems.00007-26-s0001.pdf Figures S1 to S5.

Supplemental tables msystems.00007-26-s0002.docx Tables S1 to S4.

ASM does not own the copyrights to Supplemental Material that may be linked to, or accessed through, an article. The authors have granted ASM a non-exclusive, world-wide license to publish the Supplemental Material files. Please contact the corresponding author directly for reuse.

## References

[1] Schilder AGM, Chonmaitree T, Cripps AW, Rosenfeld RM, Casselbrant ML, Haggard MP, Venekamp RP. 2016. Otitis media. Nat Rev Dis Primers 2:16063. doi:10.1038/nrdp.2016.63 27604644 PMC7097351

[2] Tamir SO, Bialasiewicz S, Brennan-Jones CG, Der C, Kariv L, Macharia I, Marsh RL, Seguya A, Thornton R. 2023. ISOM 2023 research panel 4 - diagnostics and microbiology of otitis media. Int J Pediatr Otorhinolaryngol 174:111741. doi:10.1016/j.ijporl.2023.111741 37788516

[3] Schilder AGM, Bhutta MF, Butler CC, Holy C, Levine LH, Kvaerner KJ, Norman G, Pennings RJ, Poe D, Silvola JT, Sudhoff H, Lund VJ. 2015. Eustachian tube dysfunction: consensus statement on definition, types, clinical presentation and diagnosis. Clin Otolaryngol 40:407–411. doi:10.1111/coa.12475 26347263 PMC4600223

[4] Calvo-Henriquez C, Di Corso E, Alobid I, Cantone E, Di Cesare T, Mullol J. 2023. Pathophysiological link between chronic rhinosinusitis and ear disease. Curr Allergy Asthma Rep 23:389–397. doi:10.1007/s11882-023-01072-3 37395977

[5] Santos-Cortez RLP, Ehrlich GD, Ryan AF. 2021. Editorial: otitis media genomics and the middle ear microbiome. Front Genet 12:763688. doi:10.3389/fgene.2021.763688 34712274 PMC8546293

[6] Chan CL, Wabnitz D, Bardy JJ, Bassiouni A, Wormald PJ, Vreugde S, Psaltis AJ. 2016. The microbiome of otitis media with effusion. Laryngoscope 126:2844–2851. doi:10.1002/lary.26128 27335217

[7] Chan CL, Wabnitz D, Bassiouni A, Wormald PJ, Vreugde S, Psaltis AJ. 2017. Identification of the bacterial reservoirs for the middle ear using phylogenic analysis. JAMA Otolaryngol Head Neck Surg 143:155–161. doi:10.1001/jamaoto.2016.3105 27812691

[8] Marsh RL, Aho C, Beissbarth J, Bialasiewicz S, Binks M, Cervin A, Kirkham L-AS, Lemon KP, Slack MPE, Smith-Vaughan HC. 2020. Panel 4: Recent advances in understanding the natural history of the otitis media microbiome and its response to environmental pressures. Int J Pediatr Otorhinolaryngol 130 Suppl 1:109836. doi:10.1016/j.ijporl.2019.109836 31879084 PMC7085411

[9] Lappan R, Imbrogno K, Sikazwe C, Anderson D, Mok D, Coates H, Vijayasekaran S, Bumbak P, Blyth CC, Jamieson SE, Peacock CS. 2018. A microbiome case-control study of recurrent acute otitis media identified potentially protective bacterial genera. BMC Microbiol 18:13. doi:10.1186/s12866-018-1154-3 29458340 PMC5819196

[10] Taylor SL, Papanicolas LE, Richards A, Ababor F, Kang WX, Choo JM, Woods C, Wesselingh SL, Ooi EH, MacFarlane P, Rogers GB. 2022. Ear microbiota and middle ear disease: a longitudinal pilot study of Aboriginal children in a remote south Australian setting. BMC Microbiol 22:24. doi:10.1186/s12866-022-02436-x 35026986 PMC8756658

[11] Górska-Kot A, Greenberg D, Gastoł K, Zieliński A, Givon-Lavi N. 2019. Characterization of acute otitis media otopathogens before the introduction of the pneumococcal conjugated vaccine into the national immunization program in Poland. Int J Pediatr Otorhinolaryngol 127:109666. doi:10.1016/j.ijporl.2019.109666 31491733

[12] Klein A, Tamir SO, Sorek N, Hanun G, Yeshayahu Y, Marom T. 2022. Increase in Haemophilus influenzae detection in 13-valent pneumococcal conjugate vaccine immunized children with acute otitis media. Pediatr Infect Dis J 41:678–680. doi:10.1097/INF.0000000000003561 35436266

[13] Xu F, Kong W, Peng J, Gu H, Zheng H. 2020. Analysis of main pathogenic bacteria and drug sensitivity in patients with chronic suppurative otitis media and middle ear cholesteatoma in China. Biotechnol Lett 42:1559–1566. doi:10.1007/s10529-020-02880-7 32270423

[14] Chen X, Tong MCF, Chang WT. 2024. A nomogram diagnostic model for Eustachian tube dysfunction in patients with tympanic membrane perforation. J Otolaryngol Head Neck Surg 53:19160216241293068. doi:10.1177/19160216241293068 39446836 PMC11514124

[15] Minami SB, Mutai H, Suzuki T, Horii A, Oishi N, Wasano K, Katsura M, Tanaka F, Takiguchi T, Fujii M, Kaga K. 2017. Microbiomes of the normal middle ear and ears with chronic otitis media. Laryngoscope 127:E371–E377. doi:10.1002/lary.26579 28397271

[16] Schröder S, Lehmann M, Sauzet O, Ebmeyer J, Sudhoff H. 2015. A novel diagnostic tool for chronic obstructive Eustachian tube dysfunction—the Eustachian tube score. Laryngoscope 125:703–708. doi:10.1002/lary.24922 25215457

[17] McCoul ED, Mayer SI, Tabaee A, Bedrosian JC, Marino MJ. 2019. Endoscopic evaluation of the Eustachian tube: assessment of a novel tool for grading Eustachian tube inflammation. Int Forum Allergy Rhinol 9:305–310. doi:10.1002/alr.22252 30485734

[18] Poe D, Anand V, Dean M, Roberts WH, Stolovitzky JP, Hoffmann K, Nachlas NE, Light JP, Widick MH, Sugrue JP, Elliott CL, Rosenberg SI, Guillory P, Brown N, Syms CA III, Hilton CW, McElveen JT, Singh A, Weiss RL, Arriaga MA, Leopold JP. 2018. Balloon dilation of the eustachian tube for dilatory dysfunction: a randomized controlled trial. Laryngoscope 128:1200–1206. doi:10.1002/lary.26827 28940574

[19] Kobayashi T, Morita M, Yoshioka S, Mizuta K, Ohta S, Kikuchi T, Hayashi T, Kaneko A, Yamaguchi N, Hashimoto S, Kojima H, Murakami S, Takahashi H. 2018. Diagnostic criteria for patulous Eustachian tube: a proposal by the Japan otological society. Auris Nasus Larynx 45:1–5. doi:10.1016/j.anl.2017.09.017 29153260

[20] McCoul ED, Anand VK, Christos PJ. 2012. Validating the clinical assessment of Eustachian tube dysfunction: the Eustachian tube dysfunction questionnaire (ETDQ-7). Laryngoscope 122:1137–1141. doi:10.1002/lary.23223 22374681 PMC3612400

[21] Toma S, Hopkins C. 2016. Stratification of SNOT-22 scores into mild, moderate or severe and relationship with other subjective instruments. Rhinology 54:129–133. doi:10.4193/Rhino15.072 27017484

[22] Chen Z, Hui PC, Hui M, Yeoh YK, Wong PY, Chan MCW, Wong MCS, Ng SC, Chan FKL, Chan PKS. 2019. Impact of preservation method and 16s rRNA hypervariable region on gut microbiota profiling. mSystems 4:e00271-18. doi:10.1128/msystems.00271-18 30834331 PMC6392095

[23] Chen Z, Wong PY, Ng CWK, Lan L, Fung S, Li JW, Cai L, Lei P, Mou Q, Wong SH, et al.. 2020. The intersection between oral microbiota, host gene methylation and patient outcomes in head and neck squamous cell carcinoma. Cancers (Basel) 12:3425. doi:10.3390/cancers12113425 33218162 PMC7698865

[24] Chan JYK, Ng CWK, Lan L, Fung S, Li JW, Cai L, Lei P, Mou Q, Meehan K, Lau EHL, Yeung Z, Chan KCA, Wong EWY, Chan PKS, Chen Z. 2021. Restoration of the oral microbiota after surgery for head and neck squamous cell carcinoma is associated with patient outcomes. Front Oncol 11:737843. doi:10.3389/fonc.2021.737843 34692514 PMC8527003

[25] Zhu H, Yip HC, Cheung MK, Chan HC, Ng C, Lau EHL, Yeung ZWC, Wong EWY, Leung L, Qu X, Wang D, Cai L, Chan PKS, Chan JYK, Chen Z. 2023. Convergent dysbiosis of upper aerodigestive microbiota between patients with esophageal and oral cavity squamous cell carcinoma. Int J Cancer 152:1903–1915. doi:10.1002/ijc.34460 36752573

[26] Mirarab S, Nguyen N, Warnow T. 2012. SEPP: SATé-enabled phylogenetic placement. Pac Symp Biocomput. doi:10.1142/9789814366496_0024:247-58 22174280

[27] Segata N, Izard J, Waldron L, Gevers D, Miropolsky L, Garrett WS, Huttenhower C. 2011. Metagenomic biomarker discovery and explanation. Genome Biol 12:R60. doi:10.1186/gb-2011-12-6-r60 21702898 PMC3218848

[28] Lin H, Peddada SD. 2020. Analysis of compositions of microbiomes with bias correction. Nat Commun 11:3514. doi:10.1038/s41467-020-17041-7 32665548 PMC7360769

[29] Shenhav L, Thompson M, Joseph TA, Briscoe L, Furman O, Bogumil D, Mizrahi I, Pe’er I, Halperin E. 2019. FEAST: fast expectation-maximization for microbial source tracking. Nat Methods 16:627–632. doi:10.1038/s41592-019-0431-x 31182859 PMC8535041

[30] Rohart F, Eslami A, Matigian N, Bougeard S, Lê Cao K-A. 2017. MINT: a multivariate integrative method to identify reproducible molecular signatures across independent experiments and platforms. BMC Bioinformatics 18:128. doi:10.1186/s12859-017-1553-8 28241739 PMC5327533

[31] Love MI, Huber W, Anders S. 2014. Moderated estimation of fold change and dispersion for RNA-seq data with DESeq2. Genome Biol 15:550. doi:10.1186/s13059-014-0550-8 25516281 PMC4302049

[32] Nogues JC, Pérez-Losada M, Preciado D. 2020. Review of otitis media microbiome studies: what do they tell us? Laryngoscope Investig Otolaryngol 5:936–940. doi:10.1002/lio2.460 PMC7585249 33134542

[33] Ashhurst-Smith C, Hall ST, Walker P, Stuart J, Hansbro PM, Blackwell CC. 2007. Isolation of Alloiococcus otitidis from indigenous and non-indigenous Australian children with chronic otitis media with effusion. FEMS Immunol Med Microbiol 51:163–170. doi:10.1111/j.1574-695X.2007.00297.x 17666076

[34] Jervis-Bardy J, Leong LEX, Papanicolas LE, Ivey KL, Chawla S, Woods CM, Frauenfelder C, Ooi EH, Rogers GB. 2019. Examining the evidence for an adult healthy middle ear microbiome. mSphere 4:e00456-19. doi:10.1128/mSphere.00456-19 31484741 PMC6731531

[35] Chan CL, Richter K, Wormald PJ, Psaltis AJ, Vreugde S. 2017. Alloiococcus otitidis forms multispecies biofilm with Haemophilus influenzae: effects on antibiotic susceptibility and growth in adverse conditions. Front Cell Infect Microbiol 7:344. doi:10.3389/fcimb.2017.00344 28824879 PMC5539592

[36] Patel S, Gupta RS. 2020. A phylogenomic and comparative genomic framework for resolving the polyphyly of the genus Bacillus: proposal for six new genera of Bacillus species, Peribacillus gen. nov., Cytobacillus gen. nov., Mesobacillus gen. nov., Neobacillus gen. nov., Metabacillus gen. nov. and Alkalihalobacillus gen. nov. Int J Syst Evol Microbiol 70:406–438. doi:10.1099/ijsem.0.003775 31617837

[37] Liang M, Wu W-J, Li L, Qin H, Li S-N, Zheng G-L, Hou D-M, Huang Q, Cheng L, Jie H-Q, Lu J-R, He J-C, Yang J, Wei W. 2025. Characteristics of the microbiota in the nasopharynx and nasal cavity of healthy children before and during the COVID-19 pandemic. World J Pediatr 21:836–845. doi:10.1007/s12519-025-00953-z 40742667 PMC12380981

[38] Ars B, Dirckx J. 2016. Eustachian tube function. Otolaryngol Clin North Am 49:1121–1133. doi:10.1016/j.otc.2016.05.003 27468632

[39] Alper CM, Luntz M, Takahashi H, Ghadiali SN, Swarts JD, Teixeira MS, Csákányi Z, Yehudai N, Kania R, Poe DS. 2017. Panel 2: anatomy (Eustachian tube, middle ear, and mastoid-anatomy, physiology, pathophysiology, and pathogenesis). Otolaryngol Head Neck Surg 156:S22–S40. doi:10.1177/0194599816647959 28372527

[40] Littman DR, Pamer EG. 2011. Role of the commensal microbiota in normal and pathogenic host immune responses. Cell Host Microbe 10:311–323. doi:10.1016/j.chom.2011.10.004 22018232 PMC3202012

[41] Colombo APV, Lourenço TGB, de Oliveira AM, da Costa ALA. 2025. Link between oral and gut microbiomes: the oral-gut axis. Adv Exp Med Biol 1472:71–87. doi:10.1007/978-3-031-79146-8_5 40111686

[42] Sun H, Si F, Zhao X, Li F, Qi G. 2023. The cellular redox state in Bacillus amyloliquefaciens WH1 affects biofilm formation indirectly in a surfactant direct manner. J Basic Microbiol 63:930–943. doi:10.1002/jobm.202300064 37189223

[43] Hunt BC, Xu X, Gaggar A, Swords WE. 2022. Nontypeable Haemophilus influenzae redox recycling of protein thiols promotes resistance to oxidative killing and bacterial survival in biofilms in a smoke-related infection model. mSphere 7:e00847-21. doi:10.1128/msphere.00847-21 35044805 PMC8769201

[44] Bitoun JP, Wen ZT. 2016. Transcription factor Rex in regulation of pathophysiology in oral pathogens. Mol Oral Microbiol 31:115–124. doi:10.1111/omi.12114 26172563 PMC4713358

[45] Li-Korotky HS, Lo CY, Zeng FR, Lo D, Banks JM. 2009. Interaction of phase variation, host and pressure/gas composition: pneumococcal gene expression of PsaA, SpxB, Ply and LytA in simulated middle ear environments. Int J Pediatr Otorhinolaryngol 73:1417–1422. doi:10.1016/j.ijporl.2009.07.007 19682756 PMC2891361

[46] Li B, Srivastava S, Shaikh M, Mereddy G, Garcia MR, Chiles EN, Shah A, Ofori-Anyinam B, Chu T-Y, Cheney NJ, McCloskey D, Su X, Yang JH. 2025. Bioenergetic stress potentiates antimicrobial resistance and persistence. Nat Commun 16:5111. doi:10.1038/s41467-025-60302-6 40490453 PMC12149317

[47] Mohiuddin SG, Ngo H, Orman MA. 2024. Unveiling the critical roles of cellular metabolism suppression in antibiotic tolerance. NPJ Antimicrob Resist 2:17. doi:10.1038/s44259-024-00034-7 39843626 PMC11721439

[48] Murphy EC, Frick IM. 2013. Gram-positive anaerobic cocci--commensals and opportunistic pathogens. FEMS Microbiol Rev 37:520–553. doi:10.1111/1574-6976.12005 23030831

[49] Neeff M, Broderick D, Douglas RG, Biswas K. 2024. Anaerobic bacteria dominate the cholesteatoma tissue of chronic suppurative otitis media patients. Microb Pathog 196:106935. doi:10.1016/j.micpath.2024.106935 39270753

