# Supplementary material for: The middle ear-nasopharyngeal microbiome axis associated with obstructive Eustachian tube dysfunction in chronic otitis media
Source: mSystems. 2026 May 19;11(6):e00007-26. doi: 10.1128/msystems.00007-26 (PMC13288983; doi:10.1128/msystems.00007-26)

Supplementary Figure legends

Supplementary Figure S1. Study workflow. ME, swabs from the Eustachian tube opening of the middle ear; NS, swabs from the Eustachian tube opening of the nasopharynx in surgical side; NC, swabs from the Eustachian tube opening of the nasopharynx in control side. Not all participants contributed all specimen types due to intraoperative sampling feasibility. All obtained specimens (n=78) were submitted for sequencing and included after quality control. ETD classification was performed at the ear level. Each patient contributed an operated ear and, when available, a contralateral ear that was independently assessed for obstructive ETD. Because ETD status was not assumed to be symmetric within individuals, discordant ETD phenotypes between ears were permitted. Consequently, the number of samples contributing to each ETD category differed by sampling site and side, and group sizes were not expected to be balanced.

Supplementary Figure S2. Venn graph of sample components. A total of 37 subjects and 78 samples were included for analysis. 11 subjects provided a complete set of ME, NS and NC samples; 17 of them provided both NS and NC samples; 1 provided both ME and NS sample; 1 provided both ME and NC sample; 1, 2, 4 only provided ME, NC and NS samples, respectively. ME, swabs from the Eustachian tube opening of the middle ear; NS, swabs from the Eustachian tube opening of the nasopharynx in surgical side; NC, swabs from the Eustachian tube opening of the nasopharynx in control side.

Supplementary Figure S3. The microbiota composition of various sites (ME, NS and NC) between ETD and non-ETD groups. (A) Summarized at the phylum levels. (B) Summarized at the genus level.

Supplementary Figure S4. Principal coordinates plot using Unweighted GUniFrac, Weighted GUniFrac and Bray-Curtis dissimilarity.

Supplementary Figure S5. Multi-group comparison of bacterial samples and KEGG GSEA pathway enrichment. (A) Multi-group comparison using sparse Partial Least Squares Discriminant Analysis (sPLS-DA) between NS, NC and ME samples, with or without ETD. Component-wise variance explained is indicated on the axes. (B) GSEA plot showing KEGG pathway enrichment in ME microbiota based on ranked gene expression profiles. Pathways such as ABC transporters, biosynthesis of cofactors, carbon metabolism, cell cycle, glycine, serine, and threonine metabolism and porphyrin metabolism showed differential enrichment.

Supplementary Figure S1

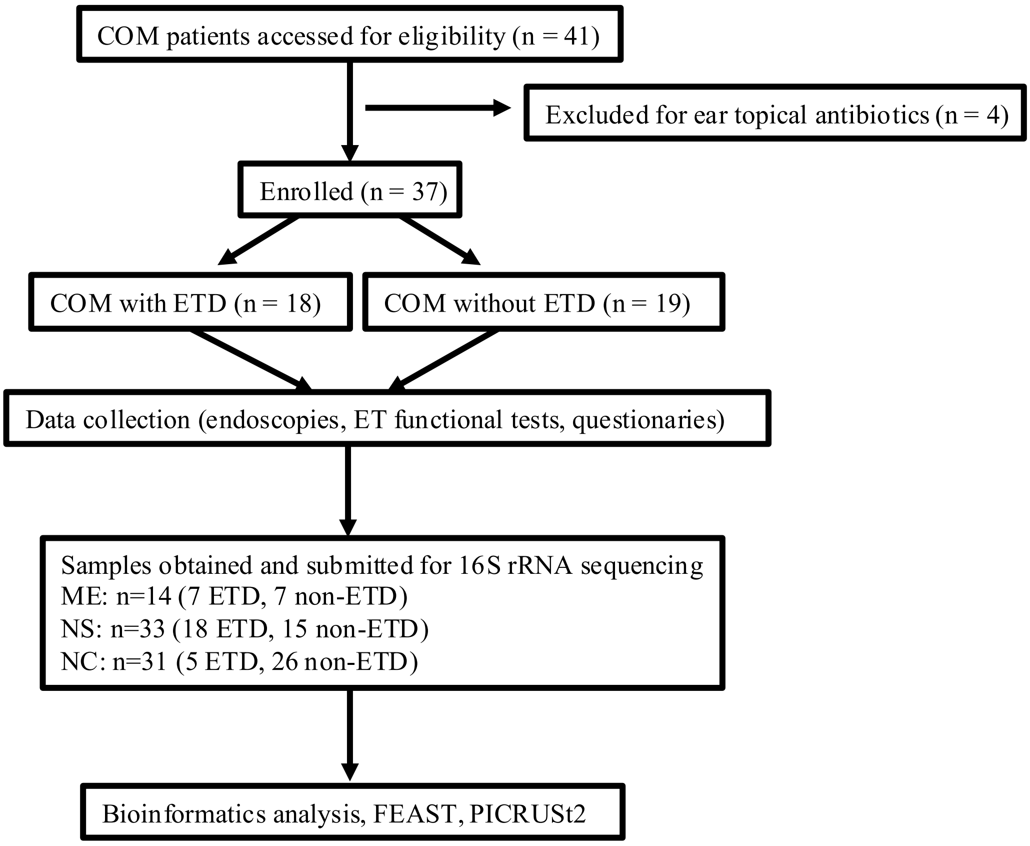

Supplementary Figure S2

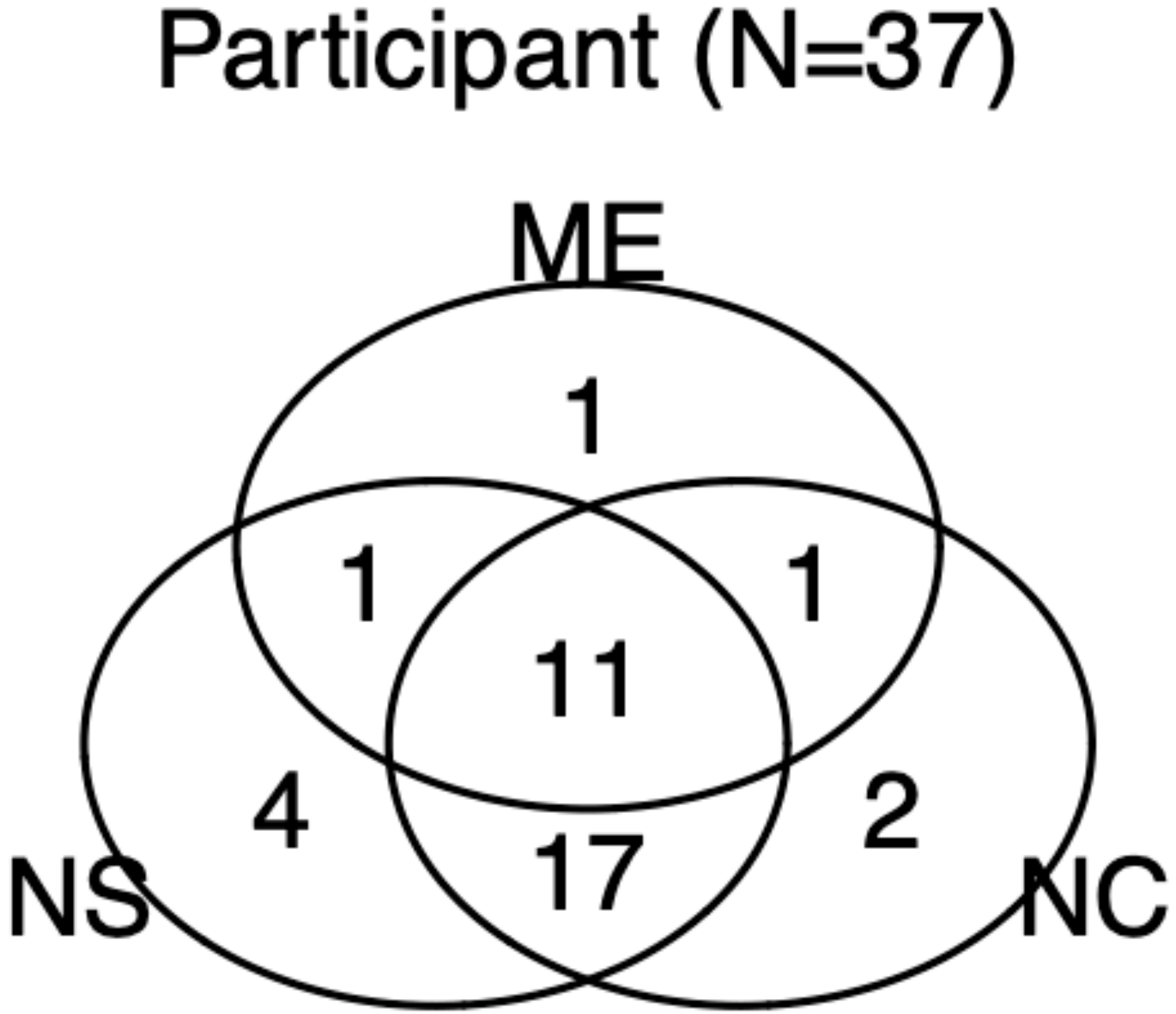

Supplementary Figure S3

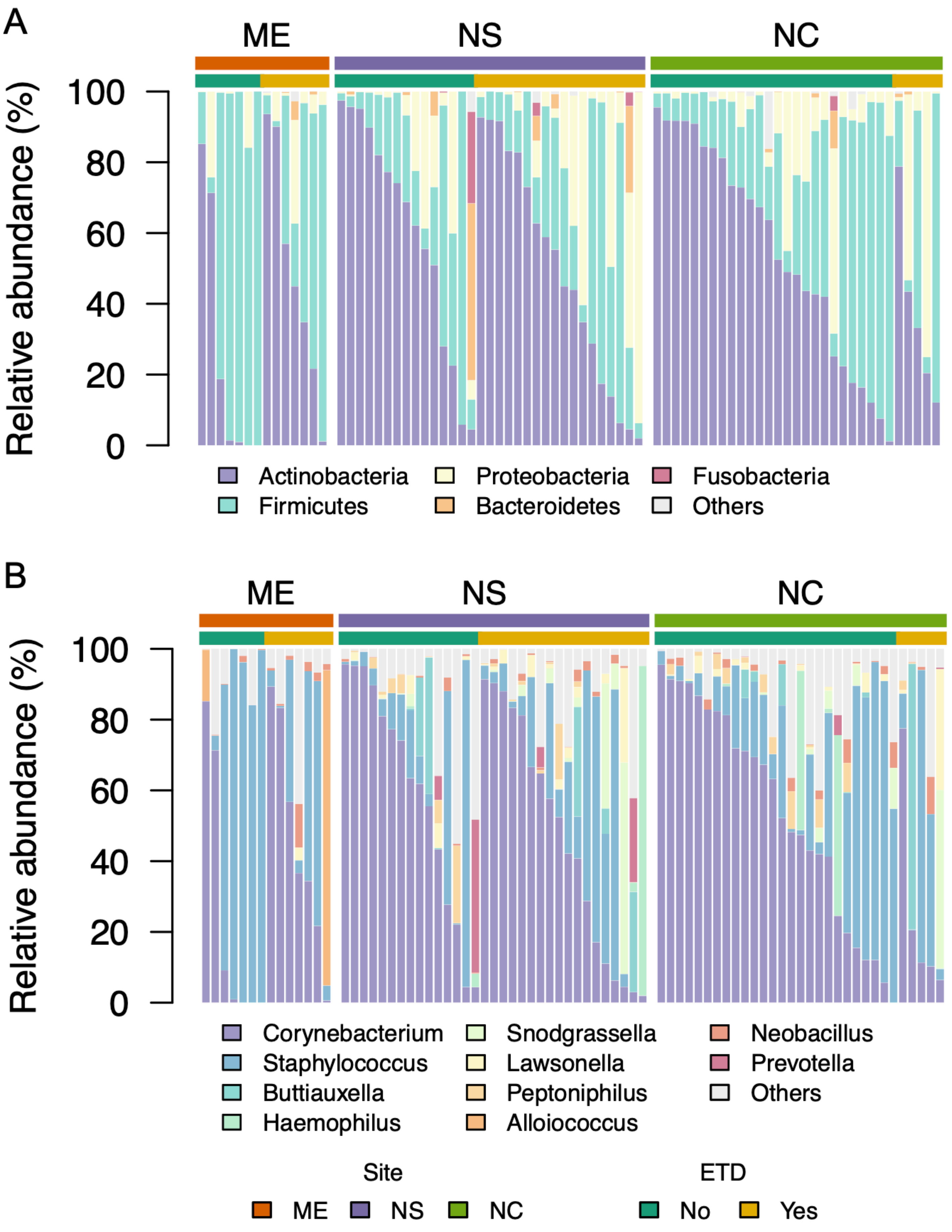

Supplementary Figure S4

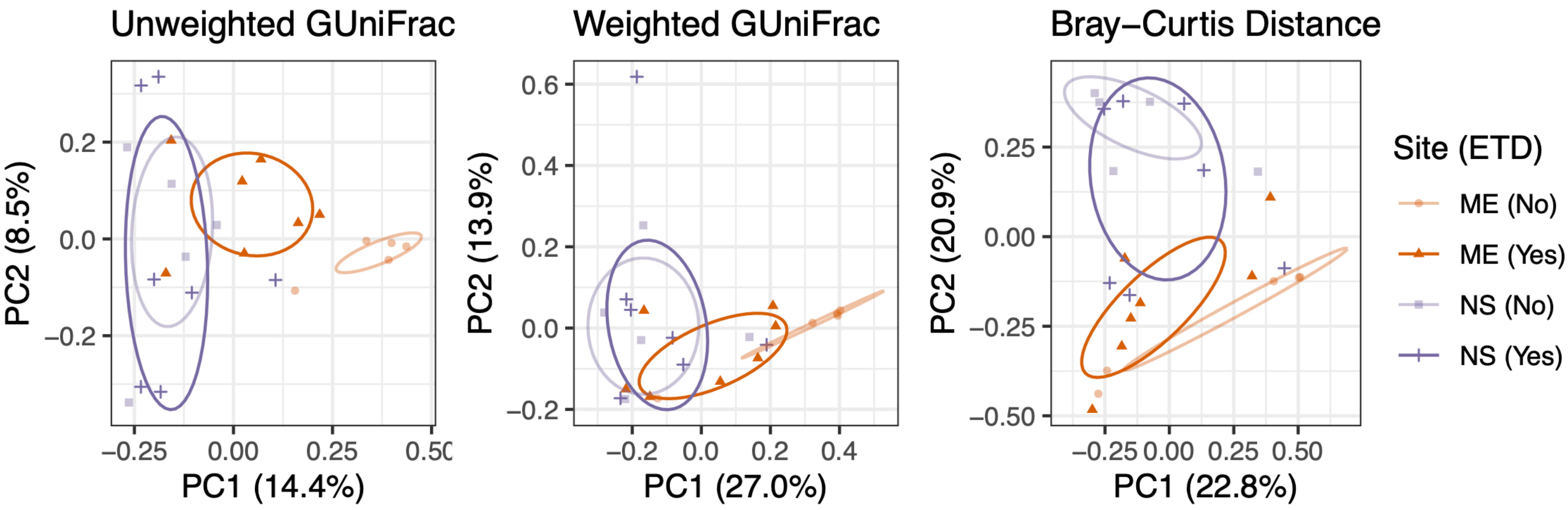

Supplementary Figure S5

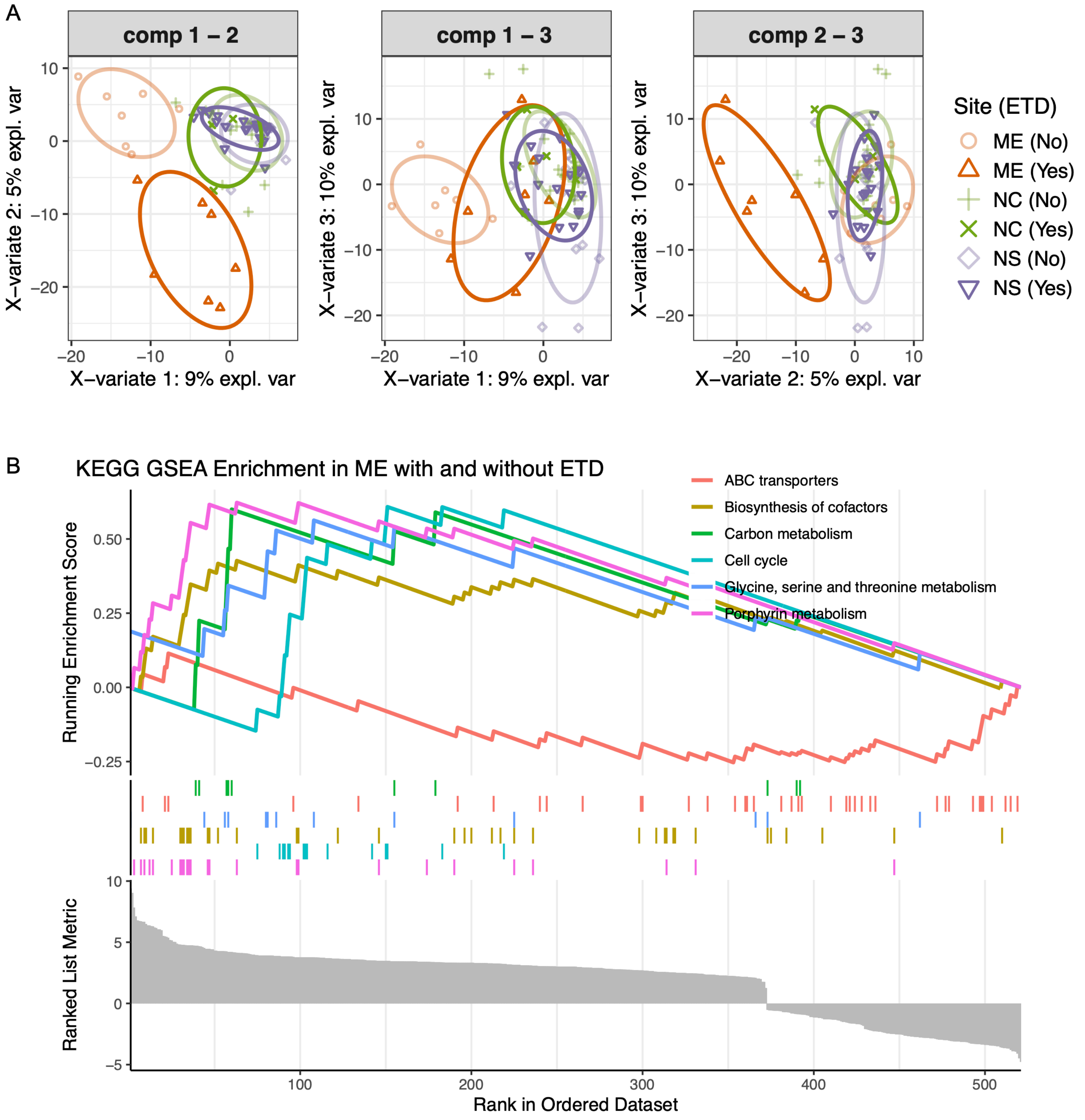

Supplement: Supplemental figures — Figures S1 to S5. [file msystems.00007-26-s0001.pdf]
