# Supplementary material for: The middle ear-nasopharyngeal microbiome axis associated with obstructive Eustachian tube dysfunction in chronic otitis media
Source: mSystems. 2026 May 19;11(6):e00007-26. doi: 10.1128/msystems.00007-26 (PMC13288983; doi:10.1128/msystems.00007-26)
Supplement: Supplemental tables — Tables S1 to S4. [file msystems.00007-26-s0002.docx]

Supplementary Table S1. Demographics and characteristics

| **Characteristic** | | **COM with ETD (N=18)** | **COM without ETD (N=19)** | ***p* value** |
| --- | --- | --- | --- | --- |
| Age (years) | | - | - | 0.898 |
| > 60 | | 38.9% (7/18) | 36.8% (7/19) |  |
| ≤ 60 | | 61.1% (11/18) | 63.2% (12/19) |  |
| Gender | | - | - | 0.642 |
| Male | | 38.9% (7/18) | 31.6% (6/19) |  |
| Female | | 61.1% (11/18) | 68.4% (13/19) |  |
| Ear side | | - | - | 0.858 |
| Left | | 44.4% (8/18) | 47.4% (9/19) |  |
| Right | | 55.6% (10/18) | 52.6% (10/19) |  |
| Onset time | | - | - | 0.556 |
| > 7 | | 27.8% (5/18) | 36.8% (7/19) |  |
| ≤ 7 | | 72.2% (13/18) | 63.2% (12/19) |  |
| Affected side | | - | - | 1.000 |
| Bilateral | | 16.7% (3/18) | 21.1% (4/19) |  |
| Unilateral | | 83.3% (15/18) | 78.9% (15/19) |  |
| CSOM | | - | - | 0.714 |
| Yes | | 72.2% (13/18) | 78.9% (15/19) |  |
| No | | 27.8% (5/18) | 21.1% (4/19) |  |
| Cholesteatoma | | - | - | 0.714 |
| Yes | | 27.8% (5/18) | 21.1% (4/19) |  |
| No | | 72.2% (13/18) | 78.9% (15/19) |  |
| Allergic rhinitis | | - | - | 1.000 |
| Yes | | 5.6% (1/18) | 10.5% (2/19) |  |
| No | | 89.5% (17/18) | 94.4% (17/19) |  |
| Chronic rhinosinusitis | - | | - | 0.002* |
| Yes | | 72.2% (13/18) | 21.1% (4/19) |  |
| No | | 27.8% (5/18) | 78.9% (15/19) |  |
| ETDQ-7 | | - | - | 0.420 |
| ≥ 25 | | 44.4% (8/18) | 31.6% (6/19) |  |
| < 25 | | 55.6% (10/18) | 68.4% (13/19) |  |
| SNOT-22 | | - | - | 0.248 |
| ≥ 40 | | 44.4% (8/18) | 26.3% (5/19) |  |
| < 40 | | 55.6% (10/18) | 73.7% (14/19) |  |

| **Characteristic** | **COM with ETD (N=18)** | **COM without ETD (N=19)** | ***p* value** |
| --- | --- | --- | --- |
| Asthma | - | - | 1.000 |
| Yes | 0% (0/18) | 5.3% (1/19) |  |
| No | 100% (18/18) | 94.7% (18/19) |  |
| Throat reflux symptoms | - | - | 1.000 |
| Yes | 27.8% (5/18) | 26.3% (5/19) |  |
| No | 72.2% (13/18) | 73.7% (14/19) |  |
| Hypertension | - | - | 1.000 |
| Yes | 11.1% (2/18) | 15.8% (3/19) |  |
| No | 88.9% (16/18) | 84.2% (16/19) |  |
| Diabetes | - | - | 0.604 |
| Yes | 5.6% (1/18) | 15.8% (3/19) |  |
| No | 94.4% (17/18) | 84.2% (16/19) |  |
| Smoking | - | - | 0.604 |
| Yes | 5.6% (1/18) | 15.8% (3/19) |  |
| No | 94.4% (17/18) | 84.2% (16/19) |  |
| Drinking | - | - | 1.000 |
| Yes | 5.6% (1/18) | 5.3% (1/19) |  |
| No | 94.4% (17/18) | 94.7% (18/19) |  |
| Eustachian tube nasopharyngeal orifice inflammation | | - | 0.001* |
| I | 16.7% (3/18) | 84.2% (16/19) |  |
| II | 55.6% (10/18) | 15.8% (3/19) |  |
| III | 16.7% (3/18) | 0% (0/19) |  |
| IV | 11.1% (2/18) | 0% (0/19) |  |
| Recurrence | - | - | 0.604 |
| Yes | 5.6% (1/18) | 15.8% (3/19) |  |
| No | 94.4% (17/18) | 84.2% (16/19) |  |

COM, chronic otitis media; ETD, Eustachian tube dysfunction; CSOM, chronic suppurative otitis media; ETDQ-7, 7-item Eustachian tube Dysfunction Questionnaire; SNOT-22, 22-item Sino-Nasal Outcome Test; **p* < 0.05.

Supplementary Table S2A. The detailed abundance of phylum of various groups.

| Phylum | All.mean | All.msd | ME.mean | ME.msd | NS.mean | NS.msd | NC.mean | NC.msd | MEvsNP\|*p* | ME_ETD\|*p* | NS_ETD\|*p* | NC_ETD\|*p* |
| --- | --- | --- | --- | --- | --- | --- | --- | --- | --- | --- | --- | --- |
| Firmicutes | 34.6884 | 3.5418 | 55.4977 | 10.5764 | 26.5884 | 4.4007 | 33.9133 | 5.3165 | 0.0582 | 0.1599 | 0.4808 | 0.7270 |
| Actinobacteria | 50.5879 | 3.6568 | 37.1994 | 9.7103 | 54.4948 | 5.6023 | 52.4753 | 5.3767 | 0.0777 | 0.1248 | 0.3201 | 0.2269 |
| Proteobacteria | 12.2067 | 2.1684 | 6.5965 | 2.5751 | 14.6774 | 3.8715 | 12.1103 | 3.3700 | 0.2309 | 0.1252 | 0.3027 | 0.5728 |
| Bacteroidetes | 1.4974 | 0.7286 | 0.4768 | 0.3839 | 2.8741 | 1.6690 | 0.4927 | 0.3457 | 0.9412 | 0.2004 | 0.6665 | 0.3788 |
| Spirochaetes | 0.0706 | 0.0653 | 0.0000 | 0.0000 | 0.1539 | 0.1539 | 0.0139 | 0.0139 | 0.5209 | - | 0.3009 | 0.7257 |
| Verrucomicrobia | 0.0082 | 0.0082 | 0.0000 | 0.0000 | 0.0194 | 0.0194 | 0.0000 | 0.0000 | 0.6641 | - | 0.3942 | - |
| Fusobacteria | 0.4908 | 0.3418 | 0.0000 | 0.0000 | 1.0325 | 0.7951 | 0.1357 | 0.1357 | 0.2422 | - | 0.8620 | 0.7257 |
| Tenericutes | 0.3825 | 0.2186 | 0.2225 | 0.1926 | 0.0230 | 0.0189 | 0.8373 | 0.5376 | 0.9092 | 0.1730 | 0.1261 | 0.1734 |
| Deinococcus-Thermus | 0.0188 | 0.0118 | 0.0071 | 0.0071 | 0.0213 | 0.0213 | 0.0215 | 0.0194 | 0.7334 | 0.3914 | 0.3942 | 0.1855 |
| Chloroflexi | 0.0061 | 0.0061 | 0.0000 | 0.0000 | 0.0143 | 0.0143 | 0.0000 | 0.0000 | 0.6641 | - | 0.3942 | - |
| Armatimonadetes | 0.0075 | 0.0075 | 0.0000 | 0.0000 | 0.0177 | 0.0177 | 0.0000 | 0.0000 | 0.6641 |  | 0.3942 |  |
| Thermotogae | 0.0352 | 0.0324 | 0.0000 | 0.0000 | 0.0831 | 0.0764 | 0.0000 | 0.0000 | 0.5209 |  | 0.2050 |  |

Supplementary Table S2B. The detailed abundance of genus of various groups.

| Genus | All.mean | All.msd | All.prevalence | ME.mean | ME.msd | NS.mean | NS.msd | NC.mean | NC.msd | MEvsNP\|*p* | ME_ETD\|*p* |
| --- | --- | --- | --- | --- | --- | --- | --- | --- | --- | --- | --- |
| Neobacillus | 1.2310 | 0.2496 | 65.3846 | 1.7508 | 0.8511 | 0.6930 | 0.1807 | 1.5690 | 0.4533 | 0.2041 | 0.0105 |
| Sphingomonas | 0.1213 | 0.0583 | 11.5385 | 0.3622 | 0.2388 | 0.0478 | 0.0478 | 0.0906 | 0.0840 | 0.0001 | 0.0236 |
| Agrobacterium | 0.0705 | 0.0378 | 6.4103 | 0.3672 | 0.1956 | 0.0000 | 0.0000 | 0.0115 | 0.0115 | 0.0002 | 0.0307 |
| Methylorubrum | 0.0915 | 0.0468 | 10.2564 | 0.4600 | 0.2385 | 0.0000 | 0.0000 | 0.0224 | 0.0224 | 0.0000 | 0.0626 |
| Staphylococcus | 26.0015 | 3.5756 | 89.7436 | 46.1140 | 11.1974 | 18.3504 | 4.5402 | 25.0631 | 5.1408 | 0.0917 | 0.1252 |
| Corynebacterium | 48.0125 | 3.7521 | 94.8718 | 34.9505 | 9.5122 | 52.1756 | 5.7437 | 49.4798 | 5.7005 | 0.0683 | 0.1580 |
| Rothia | 0.0832 | 0.0559 | 8.9744 | 0.3223 | 0.3073 | 0.0574 | 0.0229 | 0.0026 | 0.0026 | 0.8139 | 0.1730 |
| Kocuria | 0.2231 | 0.1346 | 6.4103 | 0.7836 | 0.6851 | 0.0029 | 0.0029 | 0.2043 | 0.1368 | 0.2780 | 0.1730 |
| Mesomycoplasma | 0.3585 | 0.2186 | 11.5385 | 0.2225 | 0.1926 | 0.0045 | 0.0045 | 0.7967 | 0.5388 | 0.7336 | 0.1730 |
| Thermoanaerobacterium | 0.0795 | 0.0311 | 10.2564 | 0.0704 | 0.0480 | 0.0371 | 0.0204 | 0.1288 | 0.0720 | 0.7336 | 0.1730 |
| Shinella | 0.0077 | 0.0059 | 2.5641 | 0.0428 | 0.0322 | 0.0000 | 0.0000 | 0.0000 | 0.0000 | 0.0025 | 0.1730 |
| Tumebacillus | 0.1350 | 0.0730 | 14.1026 | 0.1434 | 0.1281 | 0.0590 | 0.0237 | 0.2121 | 0.1737 | 0.7481 | 0.1730 |
| Anaerococcus | 1.0885 | 0.2665 | 50.0000 | 0.0187 | 0.0187 | 1.2767 | 0.3401 | 1.3712 | 0.5533 | 0.0006 | 0.3914 |
| Salmonella | 0.0148 | 0.0148 | 1.2821 | 0.0824 | 0.0824 | 0.0000 | 0.0000 | 0.0000 | 0.0000 | 0.0353 | 0.3914 |
| Rhodopseudomonas | 0.0490 | 0.0490 | 1.2821 | 0.2729 | 0.2729 | 0.0000 | 0.0000 | 0.0000 | 0.0000 | 0.0353 | 0.3914 |
| Stenotrophomonas | 0.0231 | 0.0215 | 2.5641 | 0.1198 | 0.1198 | 0.0037 | 0.0037 | 0.0000 | 0.0000 | 0.2345 | 0.3914 |
| Lawsonella | 1.6391 | 0.5671 | 38.4615 | 0.2568 | 0.2568 | 2.0274 | 0.8419 | 1.8500 | 1.1060 | 0.0148 | 0.3914 |
| Klebsiella | 0.0240 | 0.0144 | 3.8462 | 0.0449 | 0.0449 | 0.0117 | 0.0117 | 0.0278 | 0.0278 | 0.4941 | 0.3914 |
| Flavobacterium | 0.0149 | 0.0118 | 3.8462 | 0.0649 | 0.0649 | 0.0077 | 0.0054 | 0.0000 | 0.0000 | 0.4697 | 0.3914 |
| Calidifontibacter | 0.0269 | 0.0198 | 3.8462 | 0.0283 | 0.0283 | 0.0452 | 0.0452 | 0.0067 | 0.0067 | 0.4941 | 0.3914 |
| Deinococcus | 0.0188 | 0.0118 | 2.5641 | 0.0071 | 0.0071 | 0.0213 | 0.0213 | 0.0215 | 0.0194 | 0.7334 | 0.3914 |
| Chryseobacterium | 0.0282 | 0.0198 | 2.5641 | 0.0803 | 0.0803 | 0.0000 | 0.0000 | 0.0347 | 0.0347 | 0.2345 | 0.3914 |
| Moraxella | 0.7305 | 0.4000 | 20.5128 | 0.1948 | 0.1948 | 0.6727 | 0.6118 | 1.0339 | 0.7703 | 0.1582 | 0.3914 |
| Brucella | 0.0560 | 0.0366 | 5.1282 | 0.0126 | 0.0126 | 0.0301 | 0.0301 | 0.1031 | 0.0864 | 0.7592 | 0.3914 |
| Achromobacter | 0.3118 | 0.3107 | 1.2821 | 1.7311 | 1.7311 | 0.0025 | 0.0025 | 0.0000 | 0.0000 | 0.2345 | 0.3914 |
| Brevibacterium | 0.1628 | 0.1090 | 5.1282 | 0.4848 | 0.4848 | 0.0064 | 0.0064 | 0.1838 | 0.1682 | 0.6827 | 0.3914 |
| Sphingobium | 0.0343 | 0.0343 | 1.2821 | 0.1909 | 0.1909 | 0.0000 | 0.0000 | 0.0000 | 0.0000 | 0.0353 | 0.3914 |
| Sandaracinobacteroides | 0.0120 | 0.0099 | 2.5641 | 0.0535 | 0.0535 | 0.0000 | 0.0000 | 0.0060 | 0.0060 | 0.2345 | 0.3914 |
| Ottowia | 0.0112 | 0.0090 | 2.5641 | 0.0142 | 0.0142 | 0.0000 | 0.0000 | 0.0218 | 0.0218 | 0.2538 | 0.3914 |
| Brevundimonas | 0.0239 | 0.0158 | 3.8462 | 0.0803 | 0.0803 | 0.0135 | 0.0135 | 0.0094 | 0.0094 | 0.4697 | 0.3914 |
| Micrococcus | 0.2068 | 0.1235 | 12.8205 | 0.0360 | 0.0360 | 0.0654 | 0.0495 | 0.4344 | 0.3042 | 0.3615 | 0.3914 |
| Luteimonas | 0.0605 | 0.0605 | 1.2821 | 0.3371 | 0.3371 | 0.0000 | 0.0000 | 0.0000 | 0.0000 | 0.0353 | 0.3914 |
| Alkanindiges | 0.0134 | 0.0090 | 2.5641 | 0.0315 | 0.0315 | 0.0017 | 0.0017 | 0.0177 | 0.0177 | 0.4941 | 0.3914 |
| Knoellia | 0.0802 | 0.0612 | 5.1282 | 0.3371 | 0.3371 | 0.0272 | 0.0180 | 0.0207 | 0.0191 | 1.0000 | 0.3914 |
| Daejeonella | 0.0679 | 0.0551 | 5.1282 | 0.3316 | 0.3037 | 0.0000 | 0.0000 | 0.0211 | 0.0164 | 0.0124 | 0.4770 |
| Pseudomonas | 0.1861 | 0.0787 | 11.5385 | 0.4073 | 0.3230 | 0.0150 | 0.0150 | 0.2684 | 0.1318 | 0.2093 | 0.5938 |
| Tepidiphilus | 0.0280 | 0.0156 | 5.1282 | 0.0486 | 0.0296 | 0.0334 | 0.0334 | 0.0129 | 0.0108 | 0.0370 | 0.5938 |
| Acinetobacter | 0.4523 | 0.2073 | 21.7949 | 1.4907 | 1.0868 | 0.1677 | 0.0977 | 0.2864 | 0.1247 | 0.9438 | 0.7221 |
| Paracoccus | 0.1017 | 0.0466 | 14.1026 | 0.2517 | 0.2243 | 0.1030 | 0.0539 | 0.0326 | 0.0190 | 0.9917 | 1.0000 |
| Alloiococcus | 1.3319 | 1.1552 | 3.8462 | 7.4004 | 6.3705 | 0.0086 | 0.0086 | 0.0000 | 0.0000 | 0.0246 | 1.0000 |
| Lautropia | 0.0112 | 0.0066 | 3.8462 | 0.0000 | 0.0000 | 0.0075 | 0.0075 | 0.0201 | 0.0146 | 0.4231 | - |
| Streptococcus | 0.7862 | 0.3680 | 28.2051 | 0.0000 | 0.0000 | 0.5044 | 0.1932 | 1.4411 | 0.8975 | 0.0024 | - |
| Porphyromonas | 0.1952 | 0.1117 | 5.1282 | 0.0000 | 0.0000 | 0.3110 | 0.2270 | 0.1601 | 0.1447 | 0.2422 | - |
| Liquorilactobacillus | 0.0474 | 0.0439 | 2.5641 | 0.0000 | 0.0000 | 0.0086 | 0.0086 | 0.1102 | 0.1102 | 0.5209 | - |
| Nitrobacter | 0.0045 | 0.0036 | 1.2821 | 0.0000 | 0.0000 | 0.0107 | 0.0085 | 0.0000 | 0.0000 | 0.5209 | - |
| Clostridium | 0.0130 | 0.0130 | 1.2821 | 0.0000 | 0.0000 | 0.0308 | 0.0308 | 0.0000 | 0.0000 | 0.6641 | - |
| Veillonella | 0.2215 | 0.1579 | 5.1282 | 0.0000 | 0.0000 | 0.5167 | 0.3702 | 0.0073 | 0.0073 | 0.2901 | - |
| Treponema | 0.0708 | 0.0654 | 2.5641 | 0.0000 | 0.0000 | 0.1543 | 0.1543 | 0.0139 | 0.0139 | 0.5209 | - |
| Peptoniphilus | 1.5385 | 0.4087 | 42.3077 | 0.0000 | 0.0000 | 1.9542 | 0.8194 | 1.7906 | 0.5221 | 0.0008 | - |
| Chthoniobacter | 0.0083 | 0.0083 | 1.2821 | 0.0000 | 0.0000 | 0.0195 | 0.0195 | 0.0000 | 0.0000 | 0.6641 | - |
| Haemophilus | 2.5553 | 1.4616 | 15.3846 | 0.0000 | 0.0000 | 3.0463 | 2.8193 | 3.1866 | 2.1566 | 0.0479 | - |
| Snodgrassella | 2.5377 | 1.0933 | 29.4872 | 0.0000 | 0.0000 | 3.5868 | 2.0637 | 2.5670 | 1.6577 | 0.0095 | - |
| Peptostreptococcus | 0.0119 | 0.0094 | 2.5641 | 0.0000 | 0.0000 | 0.0070 | 0.0070 | 0.0226 | 0.0226 | 0.5209 | - |
| Fusobacterium | 0.4326 | 0.3388 | 6.4103 | 0.0000 | 0.0000 | 0.8951 | 0.7904 | 0.1357 | 0.1357 | 0.2901 | - |
| Prevotella | 1.1043 | 0.6388 | 10.2564 | 0.0000 | 0.0000 | 2.4184 | 1.4822 | 0.2042 | 0.1843 | 0.1699 | - |
| Neisseria | 0.1659 | 0.0725 | 12.8205 | 0.0000 | 0.0000 | 0.2616 | 0.1467 | 0.1389 | 0.0934 | 0.0833 | - |
| Proteus | 0.2678 | 0.2489 | 5.1282 | 0.0000 | 0.0000 | 0.5931 | 0.5880 | 0.0424 | 0.0298 | 0.3489 | - |
| Stutzerimonas | 0.0260 | 0.0260 | 1.2821 | 0.0000 | 0.0000 | 0.0614 | 0.0614 | 0.0000 | 0.0000 | 0.6641 | - |
| Acidibrevibacterium | 0.0013 | 0.0013 | 1.2821 | 0.0000 | 0.0000 | 0.0031 | 0.0031 | 0.0000 | 0.0000 | 0.6641 | - |
| Negativicoccus | 0.0339 | 0.0270 | 2.5641 | 0.0000 | 0.0000 | 0.0613 | 0.0613 | 0.0201 | 0.0201 | 0.5209 | - |
| Tsukamurella | 0.0101 | 0.0077 | 2.5641 | 0.0000 | 0.0000 | 0.0238 | 0.0182 | 0.0000 | 0.0000 | 0.5209 | - |
| Megasphaera | 0.0544 | 0.0384 | 2.5641 | 0.0000 | 0.0000 | 0.1286 | 0.0898 | 0.0000 | 0.0000 | 0.5209 | - |
| Selenomonas | 0.0726 | 0.0564 | 2.5641 | 0.0000 | 0.0000 | 0.1716 | 0.1325 | 0.0000 | 0.0000 | 0.5209 | - |
| Blastococcus | 0.0046 | 0.0046 | 1.2821 | 0.0000 | 0.0000 | 0.0000 | 0.0000 | 0.0115 | 0.0115 | 0.6641 | - |
| Terrisporobacter | 0.0054 | 0.0054 | 1.2821 | 0.0000 | 0.0000 | 0.0127 | 0.0127 | 0.0000 | 0.0000 | 0.6641 | - |
| Actirhodobacter | 0.0085 | 0.0085 | 1.2821 | 0.0000 | 0.0000 | 0.0200 | 0.0200 | 0.0000 | 0.0000 | 0.6641 | - |
| Dolosigranulum | 1.0142 | 0.2967 | 26.9231 | 0.0000 | 0.0000 | 1.8826 | 0.6262 | 0.5479 | 0.2716 | 0.0146 | - |
| Methylobacterium | 0.0166 | 0.0149 | 2.5641 | 0.0000 | 0.0000 | 0.0041 | 0.0041 | 0.0374 | 0.0374 | 0.5209 | - |
| Citrobacter | 0.0183 | 0.0127 | 3.8462 | 0.0000 | 0.0000 | 0.0054 | 0.0054 | 0.0403 | 0.0314 | 0.4231 | - |
| Qipengyuania | 0.0078 | 0.0078 | 1.2821 | 0.0000 | 0.0000 | 0.0000 | 0.0000 | 0.0197 | 0.0197 | 0.6641 | - |
| Pasteurella | 0.0209 | 0.0209 | 1.2821 | 0.0000 | 0.0000 | 0.0494 | 0.0494 | 0.0000 | 0.0000 | 0.6641 | - |
| Finegoldia | 0.5239 | 0.1861 | 26.9231 | 0.0000 | 0.0000 | 0.6021 | 0.3039 | 0.6772 | 0.3370 | 0.0048 | - |
| Shewanella | 0.0031 | 0.0031 | 1.2821 | 0.0000 | 0.0000 | 0.0074 | 0.0074 | 0.0000 | 0.0000 | 0.6641 | - |
| Filifactor | 0.0293 | 0.0244 | 2.5641 | 0.0000 | 0.0000 | 0.0563 | 0.0563 | 0.0139 | 0.0139 | 0.5209 | - |
| Leptotrichia | 0.0608 | 0.0510 | 2.5641 | 0.0000 | 0.0000 | 0.1437 | 0.1201 | 0.0000 | 0.0000 | 0.5209 | - |
| Kosakonia | 0.0113 | 0.0113 | 1.2821 | 0.0000 | 0.0000 | 0.0268 | 0.0268 | 0.0000 | 0.0000 | 0.6641 | - |
| Buttiauxella | 2.9153 | 1.2226 | 17.9487 | 0.0000 | 0.0000 | 3.8926 | 1.7667 | 3.1915 | 2.4382 | 0.0578 | - |
| Aggregatibacter | 0.0166 | 0.0166 | 1.2821 | 0.0000 | 0.0000 | 0.0000 | 0.0000 | 0.0418 | 0.0418 | 0.6641 | - |
| Campylobacter | 1.0169 | 0.5779 | 8.9744 | 0.0000 | 0.0000 | 1.7108 | 1.2162 | 0.7374 | 0.6645 | 0.1193 | - |
| Mycolicibacterium | 0.0925 | 0.0651 | 2.5641 | 0.0000 | 0.0000 | 0.1154 | 0.1154 | 0.1098 | 0.1098 | 0.5209 | - |
| Oribacterium | 0.0128 | 0.0096 | 2.5641 | 0.0000 | 0.0000 | 0.0098 | 0.0098 | 0.0219 | 0.0219 | 0.5209 | - |
| Aerococcus | 0.0034 | 0.0034 | 1.2821 | 0.0000 | 0.0000 | 0.0081 | 0.0081 | 0.0000 | 0.0000 | 0.6641 | - |
| Epilithonimonas | 0.0513 | 0.0243 | 6.4103 | 0.0000 | 0.0000 | 0.0525 | 0.0315 | 0.0731 | 0.0514 | 0.2901 | - |
| Actinomyces | 0.0040 | 0.0040 | 1.2821 | 0.0000 | 0.0000 | 0.0094 | 0.0094 | 0.0000 | 0.0000 | 0.6641 | - |
| Stomatobaculum | 0.0034 | 0.0034 | 1.2821 | 0.0000 | 0.0000 | 0.0081 | 0.0081 | 0.0000 | 0.0000 | 0.6641 | - |
| Gemella | 0.0011 | 0.0011 | 0.0000 | 0.0000 | 0.0000 | 0.0026 | 0.0026 | 0.0000 | 0.0000 | 0.6641 | - |
| Glutamicibacter | 0.0221 | 0.0157 | 3.8462 | 0.0000 | 0.0000 | 0.0000 | 0.0000 | 0.0556 | 0.0390 | 0.4231 | - |
| Chryseomicrobium | 0.0176 | 0.0105 | 3.8462 | 0.0000 | 0.0000 | 0.0000 | 0.0000 | 0.0443 | 0.0260 | 0.4231 | - |
| Aquabacterium | 0.0211 | 0.0160 | 2.5641 | 0.0000 | 0.0000 | 0.0147 | 0.0147 | 0.0374 | 0.0374 | 0.5209 | - |
| Sphingopyxis | 0.0040 | 0.0040 | 1.2821 | 0.0000 | 0.0000 | 0.0094 | 0.0094 | 0.0000 | 0.0000 | 0.6641 | - |
| Enterobacter | 0.0477 | 0.0357 | 2.5641 | 0.0000 | 0.0000 | 0.0770 | 0.0770 | 0.0380 | 0.0380 | 0.5209 | - |
| Lancefieldella | 0.0069 | 0.0069 | 1.2821 | 0.0000 | 0.0000 | 0.0163 | 0.0163 | 0.0000 | 0.0000 | 0.6641 | - |
| Pseudoprevotella | 0.0278 | 0.0197 | 3.8462 | 0.0000 | 0.0000 | 0.0657 | 0.0461 | 0.0000 | 0.0000 | 0.4231 | - |
| Lysobacter | 0.0114 | 0.0106 | 1.2821 | 0.0000 | 0.0000 | 0.0270 | 0.0251 | 0.0000 | 0.0000 | 0.5209 | - |
| Metamycoplasma | 0.0182 | 0.0129 | 2.5641 | 0.0000 | 0.0000 | 0.0186 | 0.0186 | 0.0261 | 0.0261 | 0.5209 | - |
| Brachybacterium | 0.0556 | 0.0556 | 1.2821 | 0.0000 | 0.0000 | 0.0000 | 0.0000 | 0.1400 | 0.1400 | 0.6641 | - |
| Dialister | 0.0213 | 0.0213 | 1.2821 | 0.0000 | 0.0000 | 0.0505 | 0.0505 | 0.0000 | 0.0000 | 0.6641 | - |
| Alicyclobacillus | 0.0064 | 0.0064 | 1.2821 | 0.0000 | 0.0000 | 0.0152 | 0.0152 | 0.0000 | 0.0000 | 0.6641 | - |
| Peredibacter | 0.0052 | 0.0052 | 1.2821 | 0.0000 | 0.0000 | 0.0123 | 0.0123 | 0.0000 | 0.0000 | 0.6641 | - |
| Rubrobacter | 0.0008 | 0.0008 | 0.0000 | 0.0000 | 0.0000 | 0.0019 | 0.0019 | 0.0000 | 0.0000 | 0.6641 | - |
| Escherichia | 0.0186 | 0.0120 | 3.8462 | 0.0000 | 0.0000 | 0.0439 | 0.0281 | 0.0000 | 0.0000 | 0.4231 | - |
| Amaricoccus | 0.0300 | 0.0262 | 2.5641 | 0.0000 | 0.0000 | 0.0710 | 0.0618 | 0.0000 | 0.0000 | 0.5209 | - |
| Solobacterium | 0.0038 | 0.0038 | 1.2821 | 0.0000 | 0.0000 | 0.0090 | 0.0090 | 0.0000 | 0.0000 | 0.6641 | - |
| Kaistella | 0.0027 | 0.0027 | 1.2821 | 0.0000 | 0.0000 | 0.0064 | 0.0064 | 0.0000 | 0.0000 | 0.6641 | - |
| Schaalia | 0.0047 | 0.0040 | 1.2821 | 0.0000 | 0.0000 | 0.0112 | 0.0095 | 0.0000 | 0.0000 | 0.5209 | - |
| Novosphingopyxis | 0.0012 | 0.0012 | 0.0000 | 0.0000 | 0.0000 | 0.0000 | 0.0000 | 0.0031 | 0.0031 | 0.6641 | - |
| Lysinibacillus | 0.0225 | 0.0136 | 3.8462 | 0.0000 | 0.0000 | 0.0000 | 0.0000 | 0.0566 | 0.0336 | 0.4231 | - |
| Mogibacterium | 0.0086 | 0.0071 | 2.5641 | 0.0000 | 0.0000 | 0.0041 | 0.0041 | 0.0174 | 0.0174 | 0.5209 | - |
| Parvimonas | 0.0056 | 0.0056 | 1.2821 | 0.0000 | 0.0000 | 0.0133 | 0.0133 | 0.0000 | 0.0000 | 0.6641 | - |
| Roseomonas | 0.0131 | 0.0127 | 1.2821 | 0.0000 | 0.0000 | 0.0309 | 0.0301 | 0.0000 | 0.0000 | 0.5209 | - |
| Limosilactobacillus | 0.0042 | 0.0042 | 1.2821 | 0.0000 | 0.0000 | 0.0100 | 0.0100 | 0.0000 | 0.0000 | 0.6641 | - |
| Enterocloster | 0.0038 | 0.0038 | 1.2821 | 0.0000 | 0.0000 | 0.0091 | 0.0091 | 0.0000 | 0.0000 | 0.6641 | - |
| Granulicatella | 0.3355 | 0.3107 | 5.1282 | 0.0000 | 0.0000 | 0.0456 | 0.0318 | 0.7956 | 0.7813 | 0.2422 | - |
| Taibaiella | 0.0116 | 0.0116 | 1.2821 | 0.0000 | 0.0000 | 0.0274 | 0.0274 | 0.0000 | 0.0000 | 0.6641 | - |
| Devosia | 0.0183 | 0.0183 | 1.2821 | 0.0000 | 0.0000 | 0.0000 | 0.0000 | 0.0461 | 0.0461 | 0.6641 | - |
| Peptococcus | 0.0025 | 0.0025 | 1.2821 | 0.0000 | 0.0000 | 0.0058 | 0.0058 | 0.0000 | 0.0000 | 0.6641 | - |
| Bulleidia | 0.0208 | 0.0171 | 2.5641 | 0.0000 | 0.0000 | 0.0394 | 0.0394 | 0.0104 | 0.0104 | 0.5209 | - |

Supplementary Table S3. Pairwise comparison of permutational multivariate analysis of variance (PERMANOVA) between different groups.

| Unweighted GUniFrac | Permuted *p*-value | | | | | | |
| --- | --- | --- | --- | --- | --- | --- | --- |
|  |  | ME_No | ME_Yes | NC_No | NC_Yes | NS_No | NS_Yes |
|  | ME_No |  | 0.295000 | 0.112000 | 0.197000 | 0.208000 | 0.059 |
|  | ME_Yes | 0.289211 |  | 0.750000 | 0.934000 | 0.835000 | 0.331 |
| Observed  *p*-value | NC_No | 0.111626 | 0.749255 |  | 0.784000 | 0.981000 | 0.324 |
|  | NC_Yes | 0.208096 | 0.935918 | 0.798739 |  | 0.856000 | 0.259 |
|  | NS_No | 0.218838 | 0.833495 | 0.974139 | 0.863157 |  | 0.375 |
|  | NS_Yes | 0.070651 | 0.341775 | 0.310818 | 0.272885 | 0.390224 |  |

| Weighted GUniFrac | Permuted *p*-value | | | | | | |
| --- | --- | --- | --- | --- | --- | --- | --- |
|  |  | ME_No | ME_Yes | NC_No | NC_Yes | NS_No | NS_Yes |
|  | ME_No |  | 0.168000 | 0.009000 | 0.010000 | 0.053000 | 0.025 |
|  | ME_Yes | 0.158289 |  | 0.210000 | 0.243000 | 0.502000 | 0.257 |
| Observed  *p*-value | NC_No | 0.010050 | 0.200848 |  | 0.767000 | 0.696000 | 0.718 |
|  | NC_Yes | 0.011509 | 0.224741 | 0.753585 |  | 0.905000 | 0.548 |
|  | NS_No | 0.059812 | 0.481872 | 0.697913 | 0.887995 |  | 0.512 |
|  | NS_Yes | 0.026253 | 0.235289 | 0.714068 | 0.549150 | 0.534118 |  |

| Bray–Curtis distances | Permuted *p*-value | | | | | | |
| --- | --- | --- | --- | --- | --- | --- | --- |
|  |  | ME_No | ME_Yes | NC_No | NC_Yes | NS_No | NS_Yes |
|  | ME_No |  | 0.248000 | 0.212000 | 0.282000 | 0.230000 | 0.772 |
|  | ME_Yes | 0.234659 |  | 0.017000 | 0.020000 | 0.021000 | 0.116 |
| Observed  *p*-value | NC_No | 0.204031 | 0.016941 |  | 0.840000 | 0.928000 | 0.207 |
|  | NC_Yes | 0.282357 | 0.028887 | 0.843298 |  | 0.778000 | 0.330 |
|  | NS_No | 0.214995 | 0.022689 | 0.925932 | 0.787695 |  | 0.208 |
|  | NS_Yes | 0.765178 | 0.130057 | 0.203528 | 0.309338 | 0.207760 |  |

Supplementary Table S4. Discriminative bacterial genus observed in ME samples compared with the NP samples

| Genus | LDA group | LDA score | LDA *p*-value | LDA *q*-value |
| --- | --- | --- | --- | --- |
| Sphingomonas | ME | 3.1745 | 0.0001 | 0.0001 |
| Agrobacterium | ME | 3.3014 | 0.0002 | 0.0002 |
| Methylorubrum | ME | 3.4426 | 0.0000 | 0.0000 |
| Shinella | ME | 2.9938 | 0.0023 | 0.0023 |
| Salmonella | ME | 2.8727 | 0.0325 | 0.0325 |
| Rhodopseudomonas | ME | 3.3446 | 0.0325 | 0.0325 |
| Sphingobium | ME | 3.1544 | 0.0325 | 0.0325 |
| Luteimonas | ME | 3.4194 | 0.0325 | 0.0325 |
| Daejeonella | ME | 3.2174 | 0.0119 | 0.0119 |
| Tepidiphilus | ME | 2.5423 | 0.0358 | 0.0358 |
| Alloiococcus | ME | 4.5971 | 0.0234 | 0.0234 |
| Anaerococcus | NP | 3.8242 | 0.0006 | 0.0006 |
| Lawsonella | NP | 3.9355 | 0.0145 | 0.0145 |
| Streptococcus | NP | 3.6877 | 0.0023 | 0.0023 |
| Peptoniphilus | NP | 3.9832 | 0.0008 | 0.0008 |
| Haemophilus | NP | 4.2310 | 0.0469 | 0.0469 |
| Snodgrassella | NP | 4.2091 | 0.0093 | 0.0093 |
| Dolosigranulum | NP | 3.7521 | 0.0142 | 0.0142 |
| Finegoldia | NP | 3.5151 | 0.0047 | 0.0047 |
